# Associations between COVID-19 mobility restrictions and economic, mental health, and suicide-related concerns in the US using cellular phone GPS and Google search volume data

**DOI:** 10.1371/journal.pone.0260931

**Published:** 2021-12-22

**Authors:** Catherine Gimbrone, Caroline Rutherford, Sasikiran Kandula, Gonzalo Martínez-Alés, Jeffrey Shaman, Mark Olfson, Madelyn S. Gould, Sen Pei, Marta Galanti, Katherine M. Keyes

**Affiliations:** 1 Department of Epidemiology, Columbia University, New York, NY, United States of America; 2 Department of Environmental Health Sciences, Columbia University, New York, NY, United States of America; 3 Department of Psychiatry, Columbia University, New York, NY, United States of America; Istanbul University Istanbul Faculty of Medicine: Istanbul Universitesi Istanbul Tip Fakultesi, TURKEY

## Abstract

During the COVID-19 pandemic, US populations have experienced elevated rates of financial and psychological distress that could lead to increases in suicide rates. Rapid ongoing mental health monitoring is critical for early intervention, especially in regions most affected by the pandemic, yet traditional surveillance data are available only after long lags. Novel information on real-time population isolation and concerns stemming from the pandemic’s social and economic impacts, via cellular mobility tracking and online search data, are potentially important interim surveillance resources. Using these measures, we employed transfer function model time-series analyses to estimate associations between daily mobility indicators (proportion of cellular devices completely at home and time spent at home) and Google Health Trends search volumes for terms pertaining to economic stress, mental health, and suicide during 2020 and 2021 both nationally and in New York City. During the first pandemic wave in early-spring 2020, over 50% of devices remained completely at home and searches for economic stressors exceeded 60,000 per 10 million. We found large concurrent associations across analyses between declining mobility and increasing searches for economic stressor terms (national proportion of devices at home: cross-correlation coefficient (CC) = 0.6 (p-value <0.001)). Nationally, we also found strong associations between declining mobility and increasing mental health and suicide-related searches (time at home: mood/anxiety CC = 0.53 (<0.001), social stressor CC = 0.51 (<0.001), suicide seeking CC = 0.37 (0.006)). Our findings suggest that pandemic-related isolation coincided with acute economic distress and may be a risk factor for poor mental health and suicidal behavior. These emergent relationships warrant ongoing attention and causal assessment given the potential for long-term psychological impact and suicide death. As US populations continue to face stress, Google search data can be used to identify possible warning signs from real-time changes in distributions of population thought patterns.

## Introduction

The SARS-CoV-2 pandemic and resulting illness and death related to COVID-19 are associated with deleterious effects on population mental health in the US [1–8]. There are several potential mechanisms through which the pandemic may affect mental health and suicide risk, including loss and bereavement of loved ones [9, 10], social isolation and loneliness due to quarantine and public health recommendations limiting mobility [11–13], diminished access to mental healthcare and treatment within an increasingly overwhelmed medical system [14–16], reduced ability of existing safety nets to mitigate abuse [17, 18], and unemployment and financial instability due to restrictions on business activity [19, 20]. Available data indicate increased rates of psychiatric disorders, such as depression [2, 7, 8], and exacerbated existing mental health disorders among COVID-19 patients in the first waves of the pandemic [21, 22]. Contrary to many hypotheses, however, provisional death data suggest that suicide mortality in the US did not increase during the initial pandemic outbreak [23, 24], yet there is widespread concern that rising financial insecurity and deteriorating mental health could lead to subsequent increases [25–29]. Increased suicide rates have been observed after previous pandemics [30] and recessions [31], and in other areas of the world during 2020 [32]. Using contemporary information to monitor the dynamic medium- and long-term impacts of the pandemic on economic, mental health, and suicide-related concerns is critical to guiding prevention efforts.

Accurate assessment of the evolving effects of the pandemic on economic stress, mental health, and suicide risk requires a fluid measure of population behavior over time, such as mobility (e.g. leaving one’s home or neighborhood). While cities and states passed stay-at-home orders on specific dates that can be tracked, the psychological impacts of these policies remain unclear [13] and mobility declines often preceded their passage [33, 34]. The politicization of the pandemic also spurred divisiveness over these mandates, precipitating variation in adherence to stay-at-home orders [35]. Real-time metrics of mobility can provide better insight into population movement throughout the pandemic period than can be inferred from policy implementation alone.

Estimating the near-real-time effects of COVID-19 mobility restrictions on population mental health and suicide-related concerns is challenging given long delays in the availability of survey, administrative, and vital records data. Accordingly, use of alternate sources of surveillance information such as Google search volumes has increased during the initial phases of the pandemic. Several studies have examined Google search terms as an indicator of trends in mental health outcomes and suicidal behavior, both in the US and elsewhere [36–41]. Notably, attempts to validate search volumes for suicide-related terms using suicide deaths have found heterogeneous associations [40, 41], suggesting that search volumes also capture general inquires beyond acute suicidal crises that may reflect media coverage or anomalous search trends [42–44].

Nevertheless, since the beginning of the pandemic, many studies have assessed trends in economic, mental health, and suicide-related Google search volumes across countries [26, 45–55]. Overall, search volumes for specific terms related to mental health and suicide declined in the US and elsewhere during the period in which COVID-19 cases and restrictions on gathering increased. Relative search volumes for economic stressors possibly linked to suicide risk (e.g., unemployment), however, exponentially increased in the US during the early pandemic period of March through mid-April 2020 [26]. To our knowledge, studies of search volumes in the US have not incorporated data for longer periods despite additional waves of COVID-19. Further, exploring the impact of the pandemic on Google search volumes in the US has been hindered by methodological limitations, such as selecting a single time point (e.g., date of stay-at-home order) to define complex exposures [46–48] or resorting to descriptive analyses or correlations [26, 49–51]. To date, no study has implemented time-series analyses (TSA) to explore the relationship between a dynamic proxy of the personal impacts of the pandemic, such as mobility, and economic, mental health, or suicide-related search volumes [46, 48, 54, 55].

Trends in national mental health and economic concerns may also obscure the heterogeneity in pandemic experiences in specific areas. New York City (NYC) experienced especially high case and death rates early in the pandemic, with concomitant restrictions and stay-at-home orders that led to rising unemployment rates [56–58]. It is therefore important to understand the degree to which mobility restrictions influenced search volumes within such highly affected areas where stress and trauma are anticipated to be most pronounced.

The present study investigates the relationship between cell phone mobility data and Google Health Trends (GHT) search volumes related to social and economic stress, mental health, and suicide both nationally and in the NYC designated market area (DMA) from January 2020 through January 2021, capturing the emergence and evolution of the COVID-19 pandemic across the US [59]. We conducted TSA using transfer function models [40, 60, 61] to estimate associations between mobility and search volumes in two aims. First, given that stark increases in unemployment during the past year coincided with COVID-19 lockdowns across the nation, we examined the association between trends in mobility and trends in search volumes for economic stressors as a proof of principle to establish whether a signal could be detected. We anticipated that restricted mobility would portend increases in search volumes for economic stressors, which importantly may have lasting negative effects on mental health [19, 20, 31]. Second, we assessed the impact of mobility on search volumes for mental health and suicide-related terms. If, as predicted, search volumes for economic stressors were correlated with mobility, we postulated that search volumes for mental health or suicide seeking terms might then serve as valid indicators of mental health and suicidal behavior risk. We expected mild associations between mental health and suicide-related search volumes and mobility given prior heterogenous findings on the psychological effects of lockdowns [13].

## Materials and methods

### Economic stress, mental health, and suicide-related search volumes

We extracted weekly data from the GHT application programming interface from January 6, 2019 through January 23, 2021 for primary and extended sensitivity analyses (see below for details). GHT provides the likelihood for a specified term to be queried from a location, during a period [62, 63]. This is estimated as the number of searches for the term per 10 million searches from the specified period and location. We selected a weekly, rather than daily, time resolution in order to ensure that we had sufficient search volumes across term categories. In addition to documenting national trends in search volumes, we used search volumes from the NYC DMA. DMAs correspond to geographic areas that comprise the catchment area of local television viewing [64], and are the smallest geographic unit for which GHT data are available.

### Search terms

We utilized the search term strategy outlined by Lee (2020) [41]. Terms covered a wide range of mental health and suicide-related categories including suicide neutral (e.g., “suicides”), suicide seeking (e.g., “how to kill myself”), suicide prevention (e.g., “suicide hotline”), mood and anxiety disorders (e.g., “depressed”), and psychosis (e.g., “delusion”). In order to ascertain the financial impact of the pandemic on search volumes, we divided Lee’s stressors and trauma term category into two mutually exclusive groups: economic stressors (e.g., “unemployed”) and social stressors (e.g., “lonely”, “posttraumatic stress disorder”), the latter of which we classified with mental health-related categories. Lee (2020) provides an overview of the association between these categories of search terms and suicide mortality in the US from January 2004 to December 2017 at 1 to 3-month time lags, finding heterogenous associations between search volumes and suicide mortality with further evidence of both positive and negative associations and sex differences. Given that some associations emerged for each category of searches, we used the Lee (2020) terms as they are comprehensive (S1 Table).

### Mobility metrics

Daily data on mobility both nationally and by DMA were drawn from SafeGraph [65, 66]. These data include a panel of global positioning system pins from millions of mobile devices that are measured daily and available at the census-tract level. We aggregated these data by county that we then matched to the geographic area of the NYC DMA (see S2 Table). We used two measures from SafeGraph to estimate mobility: the median time (in minutes) mobile devices were at home on a given day and the proportion of devices in a given area that did not leave their home on a given day. SafeGraph estimates ‘home’ by the common nighttime location of a device within a 153x153 meter area. We restricted the end of our study period to exclude subsequent anomalous mobility datapoints.

### Statistical analysis

We followed the methods described by Tran et al. (2017) on transfer function models [40, 61]. These methods permit assessment of the impact of our explanatory time series, mobility indicators, on our dependent time series, search volumes, while controlling for potential spurious correlations between the two. To do so, we first modeled our explanatory time series using autoregressive integrated moving average (ARIMA) models based on Box and Jenkins methods [60]. Daily mobility indicator data were averaged to match the weekly resolution of GHT data. We auto-selected the best fitting and most parsimonious models with the *forecast* package in R [67], which uses a variation of the Hyndman-Khandakar algorithm, returning the best model according to corrected Akaike information criterion. This method tests the benefit of including a seasonal component in the models using a Seasonal and Trend decomposition using Loess [67]. None of the optimal model forms selected by this method had a seasonal component. We then applied these models to the dependent time series and assessed if the two models’ residuals were correlated using the *TSA* package in R [61]. This process is termed prewhitening and any remaining correlations between models’ residuals indicate that changes in the dependent time series may be attributable to changes in the explanatory time series and vice versa.

For the primary analyses, we calculated correlations between the residuals of prewhitened explanatory and dependent time series at various time lags from January 5, 2020 through January 23, 2021 using the cross-correlation function. We visually inspected the residuals of the prewhitened time series to ensure that they met the assumptions of the normalized cross-correlation function, which approximates a time-dependent Pearson correlation coefficient [68], finding them to be independent, homoscedastic, and normally distributed [69]. Cross-correlations were assessed between time series at -14 to +14 week lags, including a 0-week lag. This range was auto-calculated based on the number of observations and time series in the data [61] and allowed for assessment of trends over several months, as recommended by Tran et al. (2017) [40]. Cross-correlation coefficients were categorized as small (.10 ≤ |r| < .30), medium (.30 ≤|r| < .50), or large (.50 ≥ |r|)) effect sizes [70]. P-values were calculated to quantify the strength of association against the null hypothesis; however they are susceptible to bias and should not be interpreted as a threshold for meaningful effects [71, 72]. A cross-correlation coefficient at a negative weekly lag (e.g. -10) indicates that changes in the *explanatory* time series lead changes in the *dependent* time series 10 weeks later. If a cross-correlation coefficient is at a positive time lag (e.g. 10), however, then it can be inferred that changes in the *dependent* time series lead changes in the *explanatory* time series 10 weeks later. A positive cross-correlation coefficient indicates that there is a direct correlation between the time series and a negative cross-correlation coefficient indicates that there is an inverse correlation between the time series. We anticipated, for example, to find larger positive cross-correlation coefficients at negative time lags between mobility indicators (explanatory) and economic stressor search volumes (dependent), meaning that decreased mobility predicted increased search volumes for terms related to economic stressors in future weeks. Cross-correlation coefficients at the 0-week lag suggest that changes in both time series were concurrent and could not be temporally disentangled.

### Sensitivity analyses

We conducted TSA sensitivity analyses using data from January 6, 2019, the earliest available month of mobility data, through January 23, 2021, in order to assess whether findings were robust to the inclusion of pre-pandemic data. Because of the extended time period, cross-correlations were auto-assessed for a broader range of weekly time lags than in primary analyses, ranging from -17 to 17 weeks.

All statistical analyses were completed in in R version 4.0.3 [73]. Data were anonymized and exempt from human subjects approval.

## Results

### Mobility metrics

**Fig 1:**
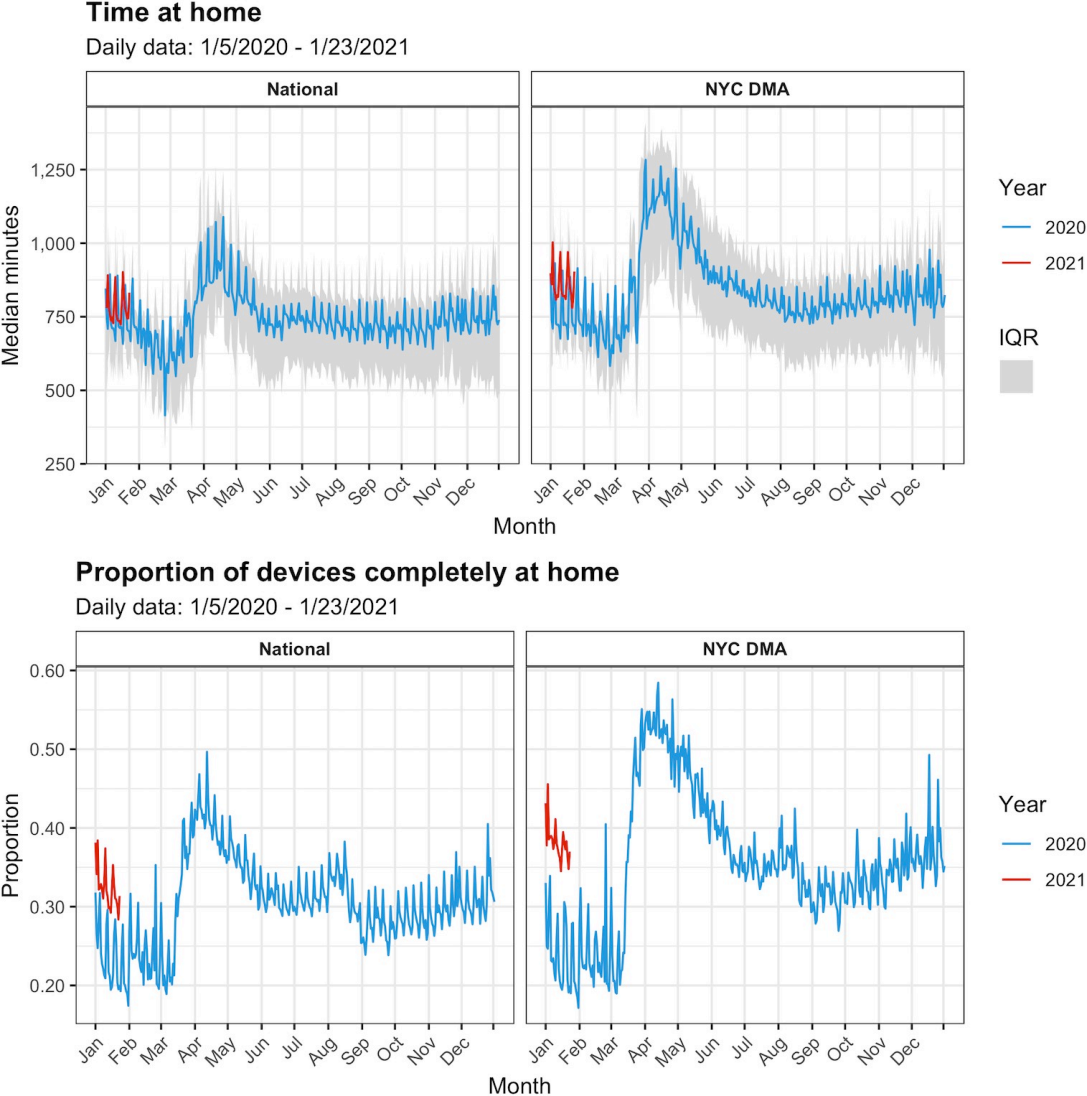
Mobility indicators over time nationally and in the NYC DMA: 2020–2021. Note: Mobility data was aggregated to the weekly-level for time-series analyses.

As illustrated in Fig 1, the proportion of devices completely at home rose sharply during March of 2020 to a daily peak in April of nearly 50% nationally and nearly 60% in the NYC DMA. During this period, the median daily amount of time that devices spent at home spiked to just over 18 hours (1,089 minutes, Interquartile Range (IQR): 859–1,245) nationally and over 21 hours (1,283 minutes, IQR: 936–1,409) in the NYC DMA. Both mobility measures declined over the course of the summer. Time at home reached its stable pre-pandemic levels, while the proportion of devices completely at home stabilized at slightly higher than its early-2020 levels. Results for the NYC DMA mirrored national trends, featuring more extreme peaks in the spring of 2020.

### Economic stress, mental health, and suicide-related search volumes

**Fig 2:**
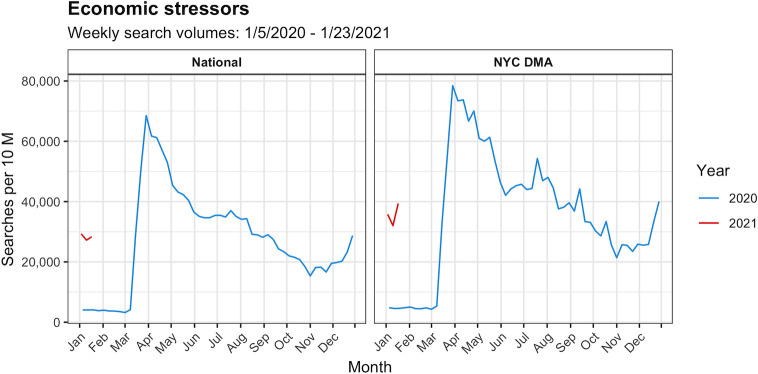
Google Health Trends search volumes for economic stressor terms over time nationally and in the NYC DMA: 2020–2021.

Fig 2 shows that search volumes (normalized per 10 million searches in a given area during a given week) for economic stressor terms rose both nationally and in the NYC DMA during March of 2020 from less than 5,000 searches per 10 million in early March to over 60,000 in late March nationally and nearing 80,000 in the NYC DMA. Search volumes for economic stressor terms then declined through the end of October, at which point they began to rise again through the beginning of 2021, coinciding with the fall and winter wave of new COVID-19 cases.

**Fig 3:**
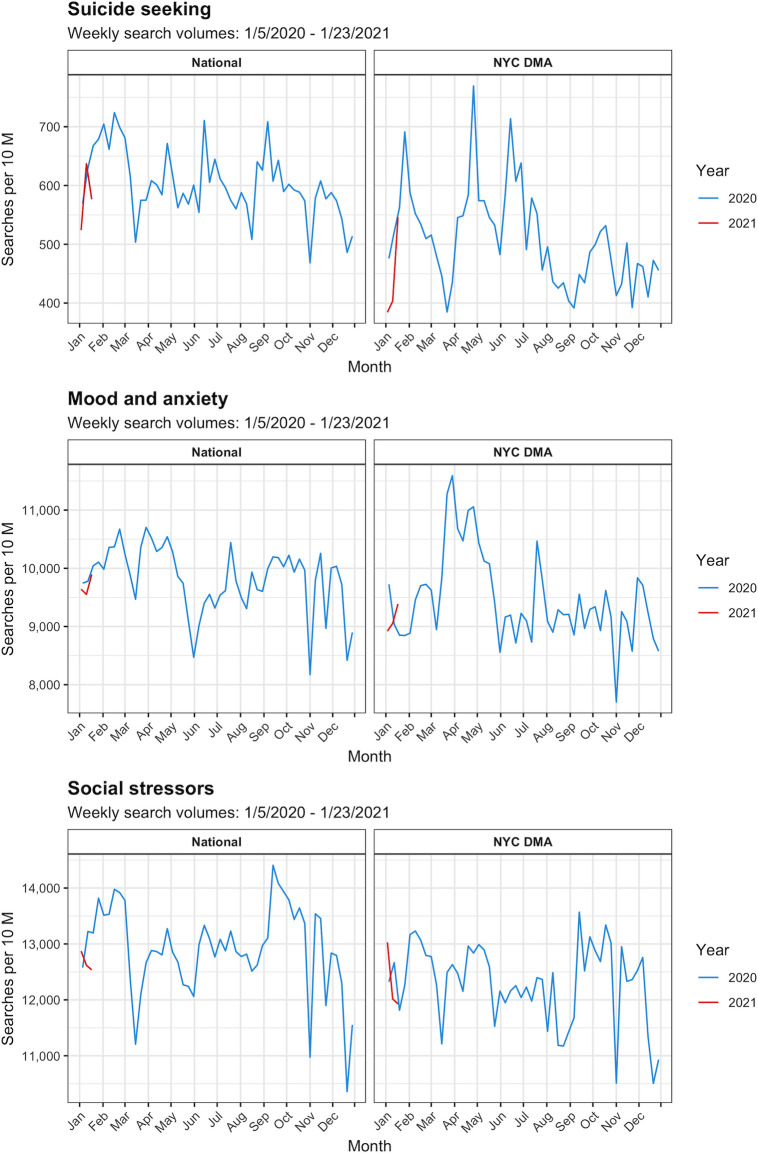
Google Health Trends search volumes for suicide seeking, mood and anxiety, and social stressor terms over time nationally and in the NYC DMA: 2020–2021.

Fig 3 highlights results for suicide seeking, mood and anxiety, and social stressor term categories (S1 Fig includes results for all other search term categories). Trends remained relatively stable during the study period for terms in the suicide seeking, suicide neutral, suicide prevention, psychosis, and mood and anxiety categories both nationally and in the NYC DMA. Similarly, social stressor terms were generally consistent over time. Temporal fluctuations in search volumes for these term categories were minor compared to those of the economic stressor terms, although all term categories exhibited a dip during the emergence of the pandemic in the middle of March 2020.

### Time series analyses (TSA)

We created heatmaps to depict TSA cross-correlation coefficients, for which numerical values and corresponding p-values can be found in S3 and S4 Tables. The algorithm selected best fit ARIMA models for each explanatory mobility time series in both primary and sensitivity analyses. Details on model parameters can be found in S5 Table. Values are reported below as (cross-correlation coefficient, *p-value*).

### Economic TSA

**Fig 4:**
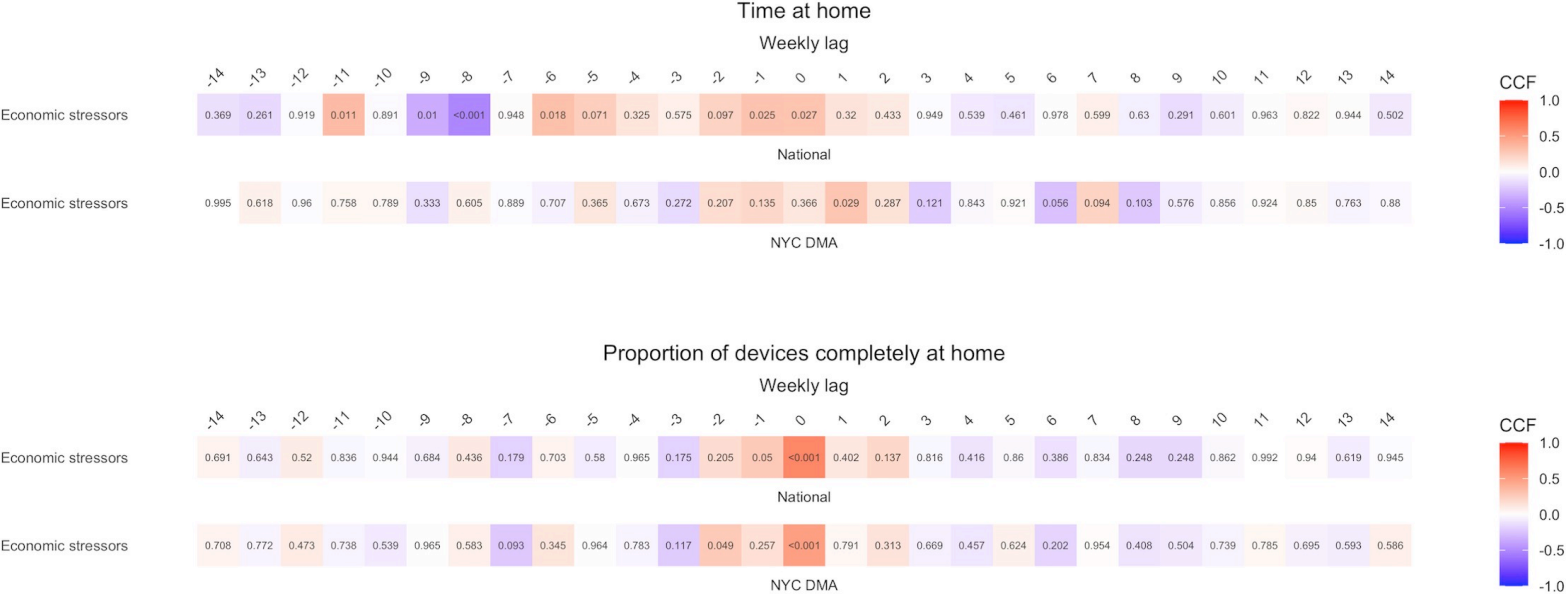
Heatmaps of cross-correlation coefficients for mobility indicators and Google Health Trends search volumes for economic stressor terms nationally and in the NYC DMA: 2020–2021. P-values listed within cells. Note on interpretation: A cross-correlation coefficient at a *negative* weekly lag indicates that changes in the *explanatory* time series lead changes in the *dependent* time series that number of weeks later. A cross-correlation coefficient at a *positive* weekly lag indicates that changes in the *dependent* time series lead changes in the *explanatory* time series that number of weeks later. Correlations at the 0-week lag suggest that changes in both time series were concurrent. A positive cross-correlation coefficient indicates that there is a direct correlation between the time series and a negative cross-correlation coefficient indicates that there is an inverse correlation between the time series.

Fig 4 presents the association between mobility and search volumes for economic stressor terms (e.g., “lost job”) that we examined as a proof of principle, given that we know that there was a strong connection between the onset of pandemic-related mobility limitations and economic stress. Larger positive cross-correlation coefficients for economic stressor terms were found at the -1 and 0-week lags for mobility indicators nationally (0.30, *0*.*027* to 0.60, <*0*.*001*), demonstrating that as mobility restrictions increased, search volumes for economic stressor terms increased both concurrently and one week later. Additionally, there were medium positive cross-correlation coefficients at weekly lags -11 (0.34, *0*.*011*) and -6 (0.32, *0*.*018*) and medium to large negative cross-correlation coefficients for weekly lags -9 (-0.35, *0*.*01*) and -8 (-0.51, <*0*.*001*) for time at home nationally. In the NYC DMA, economic stressors were positively cross-correlated with time at home at a 1-week (0.29, *0*.*029*) and with proportion of devices completely at home at -2 (0.27, *0*.*049*) and 0-week (0.49, <*0*.*001*) lags.

The largest cross-correlation coefficient occurred with proportion of devices at home nationally at a 0-week lag (0.6, <*0*.*001*). This was also the largest cross-correlation coefficient across all analyses, indicating that severe mobility restriction and economic stressor search volumes during the pandemic period were strongly correlated but temporally inextricable. Findings of some larger negative cross-correlation coefficients complicated trend interpretation; however, overall, coefficients were predominantly positive and were strongest at the 0-week lag, suggesting that search volumes for economic stressors rose concurrently with pandemic mobility restrictions both nationally and in the NYC DMA.

### Mental health and suicide TSA

**Fig 5:**
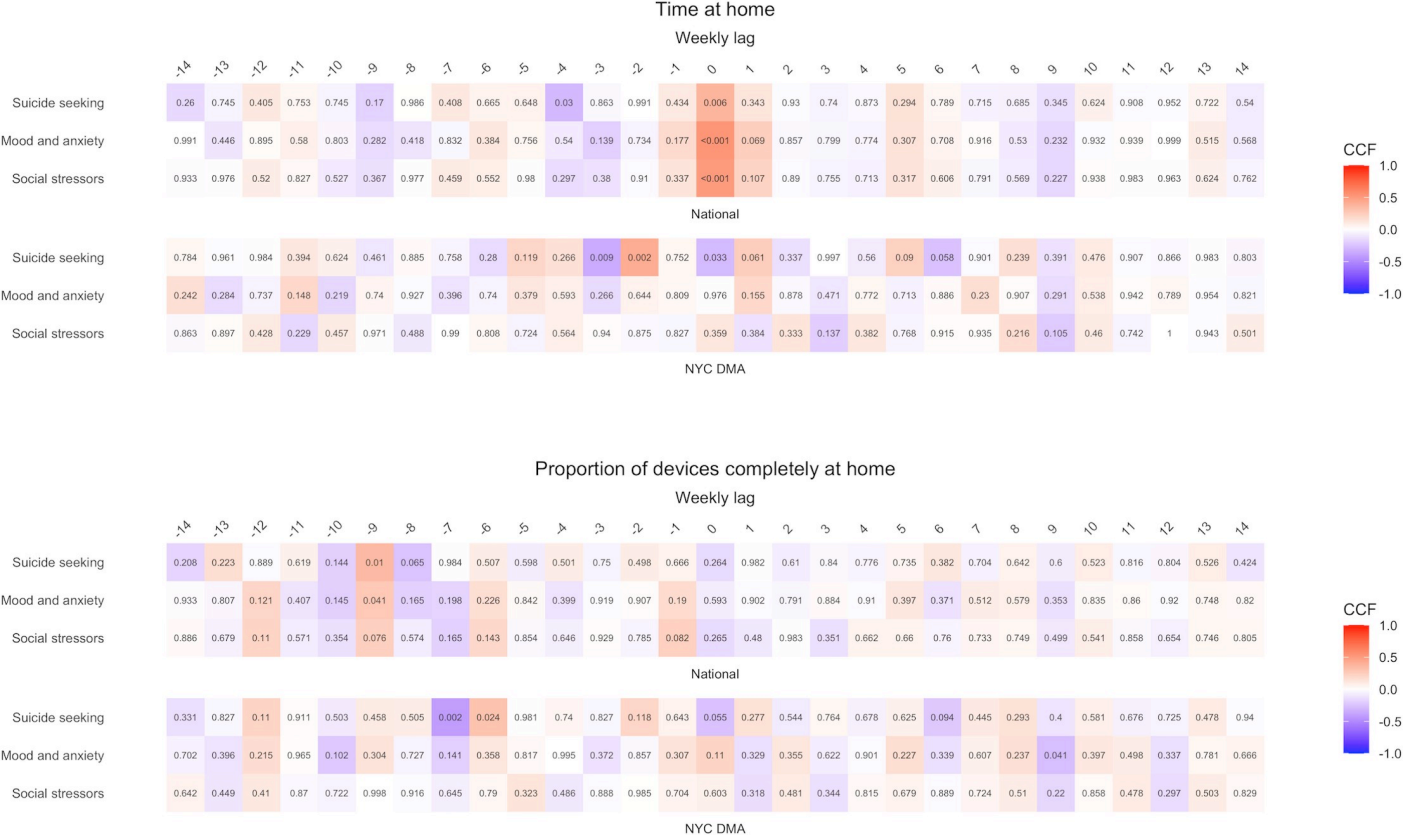
Heatmaps of cross-correlation coefficients for mobility indicators and Google Health Trends search volumes for suicide seeking, mood and anxiety, and social stressor terms nationally and in the NYC DMA: 2020–2021. P-values listed within cells. Note on interpretation: A cross-correlation coefficient at a *negative* weekly lag indicates that changes in the *explanatory* time series lead changes in the *dependent* time series that number of weeks later. A cross-correlation coefficient at a *positive* weekly lag indicates that changes in the *dependent* time series lead changes in the *explanatory* time series that number of weeks later. Correlations at the 0-week lag suggest that changes in both time series were concurrent. A positive cross-correlation coefficient indicates that there is a direct correlation between the time series and a negative cross-correlation coefficient indicates that there is an inverse correlation between the time series.

Fig 5 presents heatmaps of primary analyses cross-correlation coefficients for suicide seeking, mood and anxiety, and social stressor search volumes. S2 Fig features suicide neutral, suicide prevention, and psychosis search volumes. Trends for these search term categories diverged by location, with stronger and more consistent national associations. Nationally, a series of medium to large positive cross-correlation coefficients at a 0-week lag for time at home emerged across term categories (0.37, *0*.*006* to 0.53, <*0*.*001*), excluding suicide neutral, and were buffered by smaller positive associations at adjacent time lags. These results broadly indicate that as mobility decreased (i.e. more time at home), search volumes for terms within these categories increased at the same time. Similarly, for proportion of devices completely at home nationally, smaller positive cross-correlation coefficients were found at the -9-week lag for suicide seeking, mood and anxiety, and suicide prevention search volumes (0.28, *0*.*041* to 0.35, *0*.*01*); however, these findings were flanked by smaller negative cross-correlation coefficients which obfuscated trend interpretation. Despite differences across mobility indicators, these results suggest the declining mobility prompted increased searches for mental health and suicide-related terms during the pandemic.

Within the NYC DMA, however, there were few discernable patterns in cross-correlation coefficients for mental health and suicide-related terms across mobility indicators. Larger cross-correlation coefficients were irregularly distributed across negative and positive weekly lags and vacillated in direction. For time at home, only suicide seeking terms exhibited larger cross-correlation coefficients which alternated between positive and negative coefficients at weekly lags -3,-2, and 0 (-0.35, *0*.*009* to 0.42, *0*.*002*). An analogous pattern also arose for suicide seeking search terms with proportion of devices completely at home at the -7 (-0.41, *0*.*002*) and -6-week lags (0.3, *0*.*024*). These alternating findings denote an inexplicable and potentially immaterial relationship between mobility and searches for suicide-seeking terms in the NYC DMA.

### Sensitivity analyses

S3 Fig includes all heatmaps for sensitivity analyses with an extended study period of 2019 through 2021. As in primary analyses, we found larger, predominantly positive cross-correlation coefficients between economic stressor search volumes and mobility indicators across locations (0.21, *0*.*028* to 0.51, <*0*.*001*). These findings, however, spanned negative and positive weekly lags, signaling a bidirectional relationship between economic stress and mobility during this period.

Cross-correlation coefficients in sensitivity analyses were smaller and somewhat matched trends in primary analyses for mental health and suicide-related terms. Nationally, relatively larger positive cross-correlation coefficients for all search term categories except for suicide neutral and suicide seeking occurred at the -1-week lag between proportions of devices completely at home (0.19, *0*.*045* to 0.28, *0*.*004*). As in primary analyses, however, these positive coefficients were immediately followed by smaller negative coefficients at the 0-week lag. Similarly, a string of negative cross-correlation coefficients for time at home at a 4-week lag were bound by positive correlations at adjacent lags. Ultimately, relationships, were mixed within term categories, especially between suicide seeking terms and time at home. Within the NYC DMA, findings were similarly ambiguous with no clear trends in cross-correlation coefficients.

Overall, the majority of analyses revealed positive concurrent associations between restricted mobility and search volumes for economic stressors. From 2020 through 2021 nationally, positive relationships were found between mobility restriction and mental health and suicide-related search volumes, excluding suicide neutral, and were strongest at the 0-week lag. Similar trends were not apparent within the NYC DMA.

## Discussion

We employed mobility data documenting the proportion of cellular devices at home and time at home during the COVID-19 pandemic in the US and in New York City to assess whether restricted movement was associated with economic stress, mental health, and suicide as measured by Google Health Trends search volumes. Our research yielded two main findings. First, we found that pandemic mobility restrictions were contemporaneously linked to trajectories of online searches for terms related to economic distress across locations. Second, we found positive associations between declining mobility and mental health and suicide-related search volumes nationally during the pandemic. These results underscore the pervasiveness of pandemic-induced economic hardship and prompt further consideration of the relationships between isolation and mental health and suicide risk.

While Google data on mental health and suicide-related search volumes have been used as interim proxies for delayed epidemiological data during the pandemic [26, 45–55], to our knowledge, no studies have assessed a time-variant indicator of population behavior, such as mobility, as an exposure. Cellular mobility data provide real-time information on actual mobility restrictions [33, 34], a likely important driver of pandemic-related negative mental health outcomes [74, 75]. We found that mobility declines were most notable during the early spring of 2020 when NYC became a global center of the pandemic [76] and stay-at-home orders and social distancing recommendations were first established [77]. We also found that search volumes for economic stressor terms rapidly increased during the early stages of the pandemic, in accordance with other studies [26, 49], and in line with evidence documenting high rates of unemployment and financial hardship in response to pandemic-related economic shut-downs [78, 79]. Moreover, declining mobility was strongly correlated with concurrent and future rises in search volumes for economic stressor terms across locations, especially from 2020 through 2021, confirming our hypothesis that mobility restrictions due to travel limitations and quarantine orders would predict economic distress due to consequent economic adversity and job loss.

Having established a proof of principle, we found that search volumes for mental health and suicide-related terms declined slightly during the pandemic onset in early 2020, matching existing research [26, 46–51], and that mobility restrictions during January 2020 through January 2021 were associated with increased search volumes for these terms nationally, but not in the NYC DMA. Nationally, we identified strong concurrent relationships between mental health and suicide-related search volumes with time at home, which may be a more sensitive indicator of pandemic-induced isolation as it relates to declining psychiatric wellbeing. These findings align with longitudinal and cross-sectional studies in the US indicating that the prevalence of mental disorders and psychological distress increased during the early stages of the pandemic [1–8]. Findings of smaller correlations at heterogenous time lags that dissipated in geographically specific analyses and with the inclusion of pre-pandemic data are also consistent with evidence that lockdowns themselves have minor or mixed effects on population mental health [13, 80]. Further, provisional CDC estimates indicate that age-adjusted suicide rates during the first half of 2020 declined from those in 2019 [23]; however, emergency department visits related to suicide attempts for girls significantly increased over the course of the pandemic into 2021 [81], possibly explaining the inconsistent relationship between pandemic mobility restrictions and suicide seeking search volumes.

Foremost, our findings for economic stressor search volumes clarify the importance of financial concerns during the pandemic and highlight an opportunity to help those potentially vulnerable to medium- and long-term deteriorating mental health and increasing suicidal risk [31, 82]. Robust social safety net policies can mitigate the harms of prolonged financial distress [83, 84] and may contribute to ameliorating suicide risk [19]. Struggling individuals psychologically benefited from additional funds issued by the national government during the pandemic [85, 86] and, given the political will, governments can continue to grow and fortify the social safety net in the US [78].

The pandemic has presented many challenges to psychiatric epidemiology data collection, especially when administrative clinical service data and mortality records are lagged in their release. Our findings also contribute to the ongoing discussion surrounding the utility of Google search volumes in estimating mental health and suicide-related outcomes. Results for economic stressor, mental health, and, to some extent, suicide seeking terms matched trends in survey data. Yet attempts to assess the validity of Google search volumes, particularly in forecasting suicide deaths, [36–41] have concluded that search volumes for suicide-related terms, even when surveying granular term categories, are noisy and inconsistent indicators of suicide rates. Knipe et al. (2021, preprint) [45] analyzed the relationship between search volumes for mental health terms and mental health outcomes in the United Kingdom during the pandemic, also finding that the time series did not generally align. Ultimately, searches within the Google platform, with over 267 million unique users in the US [87], do not exactly measure population mental health outcomes and are not comparable with survey data, but instead may serve as a useful barometer for real-time population thoughts and concerns.

The extent to which Google search volumes are useful for mental health and suicide risk surveillance purposes remains unclear. For instance, search volumes for suicide-related terms often spike after a celebrity suicide death [37], which may indicate news story interest rather than increased psychological distress. Suicide deaths, however, also increase after a celebrity dies by suicide [88]. This is an example of the “Werther effect” which postulates that such increases in emulative or imitative suicide deaths among the public result from heightened news media coverage and personal identification with the celebrity [89]. Notably, Google search volumes cannot be disaggregated by social groups, such as race, although minority racial groups emerged as at growing risk of suicide mortality during the early phases of the pandemic [90, 91]. Yet despite their coarseness, spikes in search volumes for suicide-related terms may serve as early indicators of actual increases in local suicidal behavior risk, making continued surveillance warranted for public health efforts.

This study included several limitations; foremost that both cellular mobility and GHT search volumes are imperfect proxies for actual population behavior and mental health. Notably, other factors, including rates of COVID-19 illness, hospitalizations, and deaths or heightened attention to mass media, may be strongly related to mental health and suicide-related search volumes and should be explored in future analyses. Further, GHT search volumes could not be stratified by key at-risk demographic groups or adjusted for other potentially contemporaneous events that may have affected time series. We also may have been able to model and assess long-term trends and seasonality more rigorously if protracted pre-pandemic mobility data were available. Due to a lack of contemporaneous suicide mortality data, we were unable to assess the validity of suicide-related search volumes and our results may not be generalizable beyond the US.

In conclusion, we found that GHT search volumes captured real-time increases in economic distress that were temporally linked to COVID-19 pandemic mobility restrictions in the US and in New York City. Associations were also found between declining mobility and rising mental health and suicide-related search volumes nationally. Our results align with pandemic-period research exploring Google search volumes in the US and available data on unemployment, mental health, and suicide risk. As epidemiological studies strive to keep pace with the psychological impacts of the pandemic, we believe that Google search volumes may be a useful interim data source for monitoring population mental health when carefully considered.

## Supporting information

S1 Fig Google Health Trends search volumes for suicide neutral, suicide prevention, and psychosis terms over time nationally and in the NYC DMA: 2020–2021. (TIF)

S2 Fig Heatmaps of cross-correlation coefficients for mobility indicators and Google Health Trends search volumes for suicide neutral, suicide prevention, and psychosis terms nationally and in the NYC DMA: 2020–2021. P-values listed within cells. Note on interpretation: A cross-correlation coefficient at a *negative* weekly lag indicates that changes in the *explanatory* time series lead changes in the *dependent* time series that number of weeks later. A cross-correlation coefficient at a *positive* weekly lag indicates that changes in the *dependent* time series lead changes in the *explanatory* time series that number of weeks later. Correlations at the 0-week lag suggest that changes in both time series were concurrent. A positive cross-correlation coefficient indicates that there is a direct correlation between the time series and a negative cross-correlation coefficient indicates that there is an inverse correlation between the time series.
(TIF)

S3 Fig Heatmaps of cross-correlation coefficients for mobility indicators and Google Health Trends search volumes for all search term categories nationally and in the NYC DMA: 2019–2021. P-values listed within cells. Note on interpretation: A cross-correlation coefficient at a *negative* weekly lag indicates that changes in the *explanatory* time series lead changes in the *dependent* time series that number of weeks later. A cross-correlation coefficient at a *positive* weekly lag indicates that changes in the *dependent* time series lead changes in the *explanatory* time series that number of weeks later. Correlations at the 0-week lag suggest that changes in both time series were concurrent. A positive cross-correlation coefficient indicates that there is a direct correlation between the time series and a negative cross-correlation coefficient indicates that there is an inverse correlation between the time series.
(TIF)

S1 Table Google search terms sourced from Lee (2020) [41]. (PDF)

S2 Table Counties matched to the New York City designated market area. FIPS = Federal Information Processing Standards.
(PDF)

S3 Table Cross-correlation coefficients and p-values for mobility indicators Google Health Trends search volumes for all search term categories at weekly lags: 2020–2021. P-value in parenthesis.
(PDF)

S4 Table Cross-correlation coefficients and p-values for mobility indicators and Google Health Trends search volumes for all search term categories at weekly lags: 2019–2021. P-value in parenthesis.
(PDF)

S5 Table Fitted model parameters to mobility indicators nationally and in the NYC DMA. ARIMA = autoregressive integrated moving average model. Non-seasonal ARIMA parameters = (p,d,q). p = autoregressive model order, d = degree of differencing, q = moving average model order.
(PDF)

S1 Dataset (CSV)
