# Supplementary figures and images for: Associations between COVID-19 mobility restrictions and economic, mental health, and suicide-related concerns in the US using cellular phone GPS and Google search volume data
Source: PLoS One. 2021 Dec 22;16(12):e0260931. doi: 10.1371/journal.pone.0260931 (PMC8694413; doi:10.1371/journal.pone.0260931)

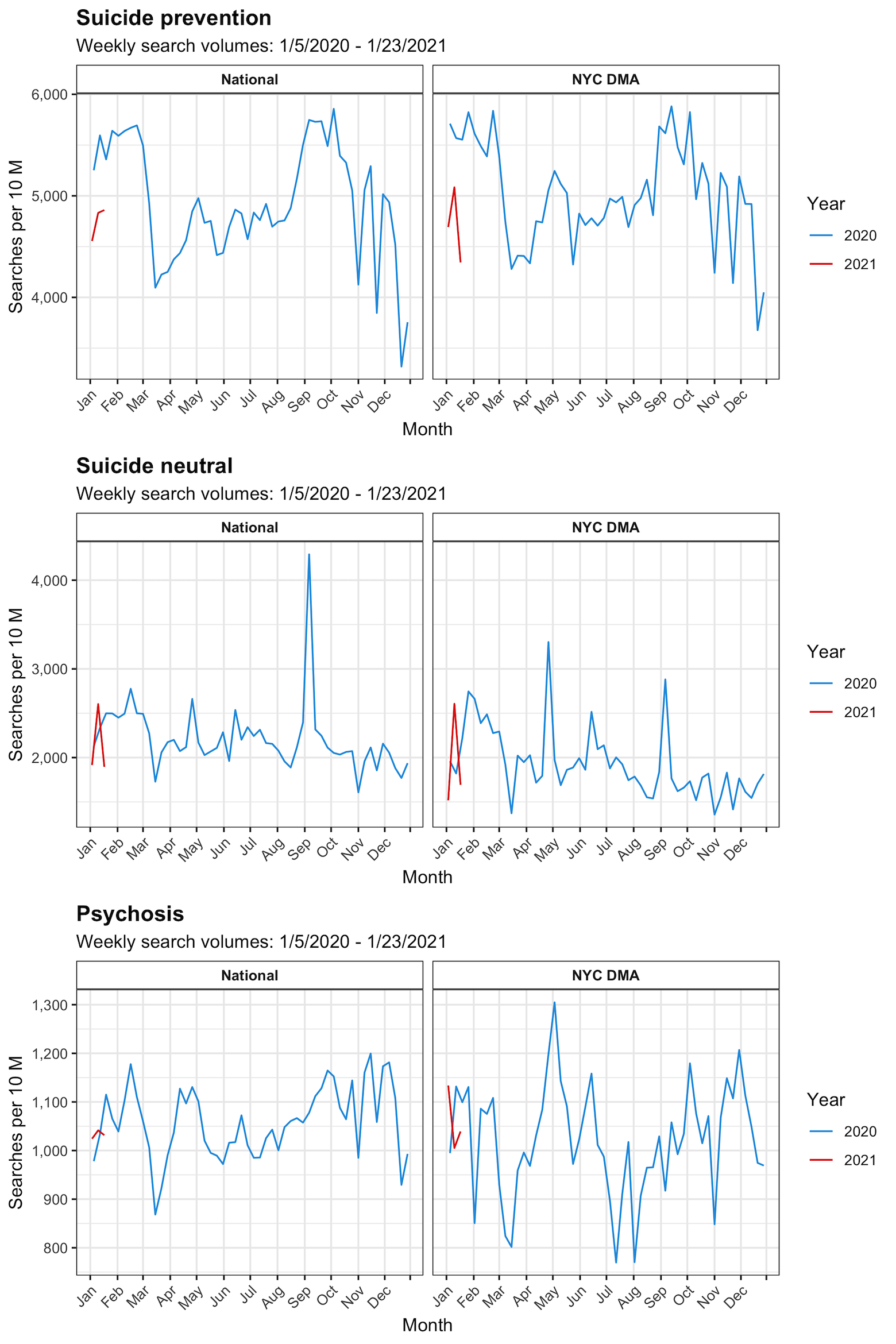

Supplement: S1 Fig — (TIF) [file pone.0260931.s001.tif]

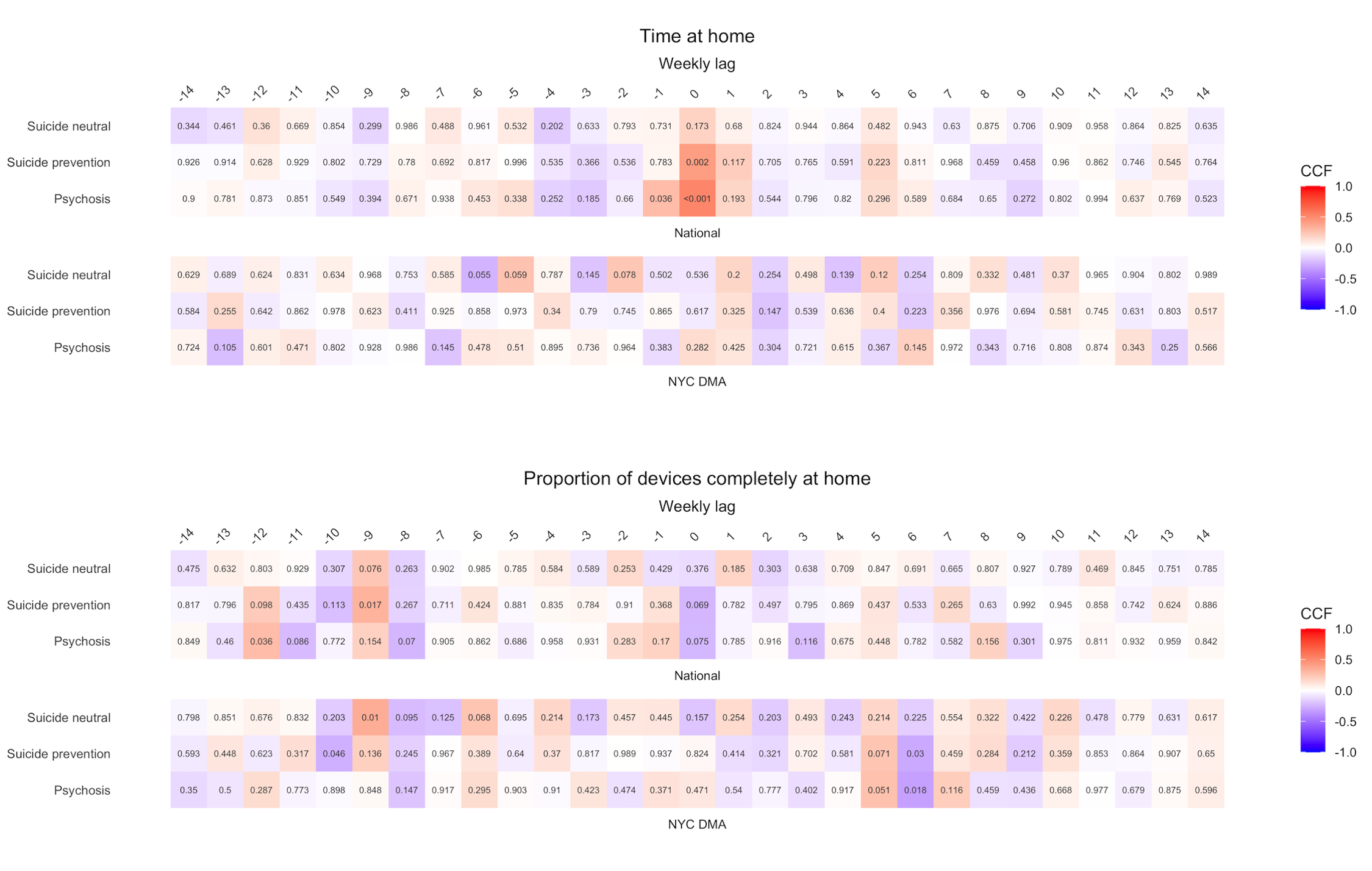

Supplement: S2 Fig — P-values listed within cells. Note on interpretation: A cross-correlation coefficient at a negative weekly lag indicates that changes in the explanatory time series lead changes in the dependent time series that number of weeks later. A cross-correlation coefficient at a positive weekly lag indicates that changes in the dependent time series lead changes in the explanatory time series that number of weeks later. Correlations at the 0-week lag suggest that changes in both time series were concurrent. A positive cross-correlation coefficient indicates that there is a direct correlation between the time series and a negative cross-correlation coefficient indicates that there is an inverse correlation between the time series. (TIF) [file pone.0260931.s002.tif]

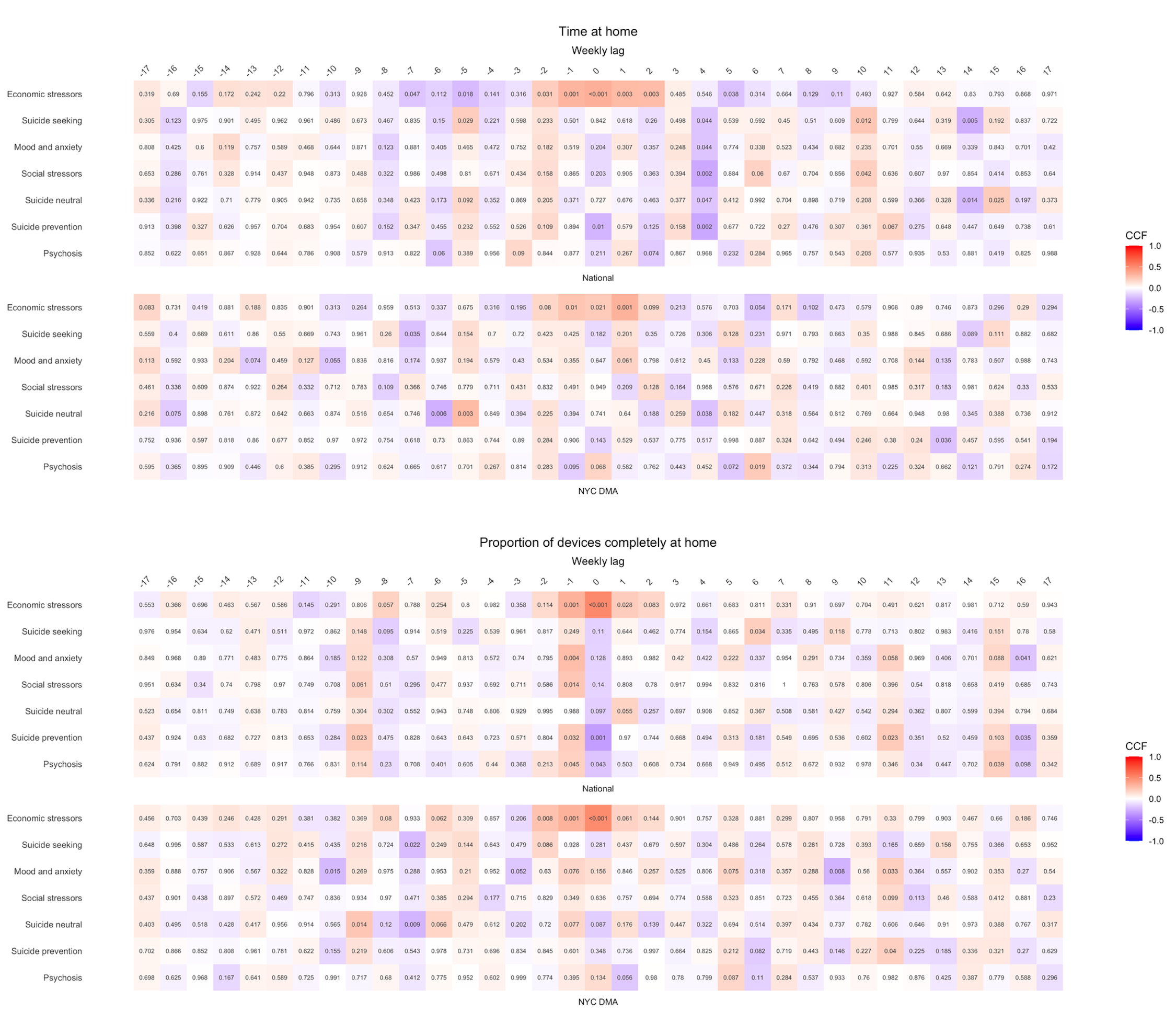

Supplement: S3 Fig — P-values listed within cells. Note on interpretation: A cross-correlation coefficient at a negative weekly lag indicates that changes in the explanatory time series lead changes in the dependent time series that number of weeks later. A cross-correlation coefficient at a positive weekly lag indicates that changes in the dependent time series lead changes in the explanatory time series that number of weeks later. Correlations at the 0-week lag suggest that changes in both time series were concurrent. A positive cross-correlation coefficient indicates that there is a direct correlation between the time series and a negative cross-correlation coefficient indicates that there is an inverse correlation between the time series. (TIF) [file pone.0260931.s003.tif]
