# Supplementary material for: Associations between COVID-19 mobility restrictions and economic, mental health, and suicide-related concerns in the US using cellular phone GPS and Google search volume data
Source: PLoS One. 2021 Dec 22;16(12):e0260931. doi: 10.1371/journal.pone.0260931 (PMC8694413; doi:10.1371/journal.pone.0260931)
Supplement: S1 Table — (PDF) [file pone.0260931.s004.pdf]

Category	Search term (short form)	Original search term
Suicide-neutral	*suicide - squad*	suicide - squad
	suicides	suicides + suicidal
	suicide idea	suicide idea + suicide ideas + suicidal idea + suicidal ideas
	suicide ideation	suicide ideation + suicide ideations + suicidal ideation + suicidal ideations
	suicide thought	suicide thought + suicide thoughts + suicidal thought + suicidal thoughts
	suicide plan	suicide plan + suicide plans + suicidal plan + suicidal plans
	suicide attempt	suicide attempt + suicide attempts + suicidal attempt + suicidal attempts
	suicidality	suicidality
	suicide survivor	suicide survivor + suicide survivors
	teen suicide	teen suicide + teens suicide + teen suicides + teens suicides
	adolescent suicide	adolescent suicide + adolescents suicide + adolescent suicides + adolescents suicides
	child suicide	child suicide + child suicides + children suicide + children suicides
	adult suicide	adult suicide + adult suicides + adults suicides+ adults suicides
	elderly suicide	elderly suicide + elderly suicides + elderlies suicide + elderlies suicides
	celebrity suicide	celebrity suicide + celebrities suicide + celebrity suicides + celebrities suicides
Suicide-seeking	*commit suicide*	commit suicide + committing suicide
	"*want to die*"	"want to die" + "wanna die"
	"*how to kill yourself*"	"how to kill yourself" + "how to kill myself"
	kill myself	kill yourself + killing yourself + kill myself + killing myself
	successful suicide	successful suicide + success suicide + successful suicides + success suicides
	effective suicide	effective suicide + effective suicides
	suicide chat	suicide chat + suicide chatting + suicide chatroom
	suicide forum	suicide forum
	how suicide	how suicide
	suicide way	suicide way + suicide ways
	suicide method	suicide method + suicide methods
	suicide strategy	suicide strategy + suicide strategies

	suicide means	suicide mean + suicide means
	assisted suicide	assisted suicide + assisted suicides
	painless suicide	painless suicide + painless suicides
	suicide poison	suicide poison + suicide poisons + suicide poisoning
	suicide overdose	suicide overdose + suicide overdoses + suicide overdosing
	overdose myself	overdose myself + overdose yourself + overdosing myself + overdosing yourself
	suicide gun	suicide gun + suicide guns + suicide firearm + suicide firearms
	shoot myself	shoot myself + shoot yourself + shooting myself + shooting yourself
	shoot suicide	shoot suicide + shooting suicide + shoot suicides + shooting suicides
	suicide jump	jump suicide + jumping suicide + jump suicides + jumping suicides
	suicide hang	hang suicide + hanging suicide + hang suicides + hanging suicides
	hang myself	hang myself + hang yourself + hanging myself + hanging yourself
	suicide note	suicide note + suicide notes + suicide letter + suicide letters
	suicide site	suicide site + suicide sites + suicide location + suicide locations + suicide spot + suicide spots
Suicide-prevention	*suicide help*	suicide help + suicide helping
	suicide hotline	suicide hotline + crisis hotline + lifeline
	suicide prevention	prevent suicide + preventing suicide + prevention suicide + preventive suicide
	suicide treatment	treat suicide + treating suicide + treatment suicide + treatments suicide
	suicide medication	suicide medication + suicide medications
	suicide clinic	suicide clinic + suicide hospital + suicide clinics + suicide hospitals
	suicide psychiatrist	suicide psychiatrist + suicide psychiatrists
	suicide psychologist	suicide psychologist + suicide psychologists
	suicide therapist	suicide therapist + suicide therapists + suicide therapy
	suicide counseling	suicide counseling + suicide counselor
	suicide psychotherapy	suicide psychotherapy + suicide psychotherapist + suicide psychotherapists
	psychiatrist	psychiatrist + psychiatrists
	psychologist	psychologist + psychologists

	psychotherapy	psychotherapy + psychotherapist + psychotherapists
	relaxation	relaxation + relaxing + relaxed
	"*cognitive behavioral therapy*"	"cognitive behavioral therapy" + "cognitive behavioral therapist" + "CBT"
	"*mental health*"	"mental health" + "behavioral health"
	antidepressant	antidepressant + antidepressants
Psychosis	*schizophrenia*	schizophrenia + schizophrenic
	schizophreniform	schizophreniform
	schizoaffective	schizoaffective
	psychosis	psychosis + psychotic
	delusion	delusion + delusions + delusional
	hallucination	hallucination + hallucinations + hallucinatory
Mood/anxiety	*bipolar*	bipolar
	"*bipolar disorder*"	"bipolar disorder" + "bipolar affective" + "bipolar I" + "bipolar II" + "manic depressive"
	mania	mania + "manic episode" + "manic episodes"
	hypomania	hypomania + "hypomanic episode" + "hypomanic episodes"
	"*bipolar depression*"	"bipolar depression"
	"*mood disorder*"	"mood disorder" + "mood disorders"
	hopeless	hopeless + hopelessness + despair
	depression	depression + depressions + depressive + depressed
	"*major depressive disorder*"	"major depression" + "major depressive disorder" + "unipolar depression" + "MDD"
	nostalgia	nostalgic + nostalgia
	anxiety	anxious + anxiety
	"*generalized anxiety disorder*"	"generalized anxiety disorder"
	"*anxiety disorder*"	"anxiety disorder" + "anxiety disorders"
	"*panic attack*"	"panic attack" + "panic attacks"
	"*panic disorder*"	"panic disorder" + "panic disorders"
	phobia	phobia + phobias
	agitation	agitation + agitated
	"*sleep disorder*"	"sleep disorder" + "sleep disorders"
	sleep problem	sleep problem + sleep problems + sleeping problem + sleeping problems

	sleep trouble	sleep trouble + sleep troubles + sleeping trouble + sleeping troubles
	"*cannot sleep*"	"cannot sleep" + "can't sleep" + "unable to sleep"
	"*cannot fall asleep*"	"cannot fall asleep" + "can't fall asleep" + "unable to fall asleep"
	insomnia	insomnia + insomniac
Social stressor	*stress*	stress + stressed + stressful
	burnout	burnout + "burn out" + "burnt out"
	fatigue	fatigue + fatigued
	distress	distress + distressed
	divorce	divorce + divorced
	separation	separation + separating + separated
	breakup	breakup + "break up" + "breaking up"
	"*social isolation*"	"social isolation" + "socially isolated" + "isolated socially"
	"*social support*"	"social support" + "social supports"
	loneliness	lonely + loneliness + lonesome
	relationship problem	relationship problem + relationship problems + relationship trouble + relationship troubles
	interpersonal problem	interpersonal problem + interpersonal problems + interpersonal trouble + interpersonal troubles
	trauma	trauma + traumas + traumatic
	"*posttraumatic stress disorder*"	"post-traumatic stress disorder" + "posttraumatic stress disorder" + PTSD
	abuse	abuse + abusing + abused + abusive
	sexual abuse	sexual abuse + sexually abusing + sexually abused + sexually abusive
	verbal abuse	verbal abuse + verbally abusing + verbally abused + verbally abusive
	physical abuse	physical abuse + physically abusing + physically abused + physically abusive
	mental abuse	mental abuse + mentally abusing + mentally abused + mentally abusive
Economic stressor	*unemployment*	unemployed + unemployment + jobless + joblessness
	"*laid off*"	"laid off" + layoff + layoffs
	fired job	fired job + fired company + fired work + fired firm
	lose job	lose job + lost job + losing job
