# Supplementary material for: Associations between COVID-19 mobility restrictions and economic, mental health, and suicide-related concerns in the US using cellular phone GPS and Google search volume data
Source: PLoS One. 2021 Dec 22;16(12):e0260931. doi: 10.1371/journal.pone.0260931 (PMC8694413; doi:10.1371/journal.pone.0260931)
Supplement: S2 Table — FIPS = Federal Information Processing Standards. (PDF) [file pone.0260931.s005.pdf]

State	County	FIPS Code
CT	Fairfield	9001
NJ	Bergen	34003
NJ	Essex	34013
NJ	Hudson	34017
NJ	Hunterdon	34019
NJ	Middlesex	34023
NJ	Monmouth	34025
NJ	Morris	34027
NJ	Ocean	34029
NJ	Passaic	34031
NJ	Somerset	34035
NJ	Sussex	34037
NJ	Union	34039
NJ	Warren	34041
NY	Bronx	36005
NY	Dutchess	36027
NY	Kings	36047
NY	Nassau	36059
NY	New York	36061
NY	Orange	36071
NY	Putnam	36079
NY	Queens	36081
NY	Richmond	36085
NY	Rockland	36087
NY	Suffolk	36103
NY	Sullivan	36105
NY	Ulster	36111
NY	Westchester	36119
PA	Pike	42103
