# Supplementary material for: Associations between COVID-19 mobility restrictions and economic, mental health, and suicide-related concerns in the US using cellular phone GPS and Google search volume data
Source: PLoS One. 2021 Dec 22;16(12):e0260931. doi: 10.1371/journal.pone.0260931 (PMC8694413; doi:10.1371/journal.pone.0260931)
Supplement: S3 Table — P-value in parenthesis. (PDF) [file pone.0260931.s006.pdf]

Primary Analyses: January 5, 2020 - January 23, 2021

**Proportion of devices at home**		**Weekly lag**																												
**Region**	**Term Category**	-14	-13	-12	-11	-10	-9	-8	-7	-6	-5	-4	-3	-2	-1	0	1	2	3	4	5	6	7	8	9	10	11	12	13	14
National	suicide neutral	-0.1 (0.475)	0.06 (0.632)	0.03 (0.803)	0.01 (0.929)	-0.14 (0.307)	0.24 (0.076)	-0.15 (0.263)	-0.02 (0.902)	0 (0.985)	0.04 (0.785)	0.07 (0.584)	-0.07 (0.589)	0.15 (0.253)	-0.11 (0.429)	-0.12 (0.376)	0.18 (0.185)	-0.14 (0.303)	-0.06 (0.638)	0.05 (0.709)	0.03 (0.847)	0.05 (0.691)	-0.06 (0.665)	0.03 (0.807)	-0.01 (0.927)	-0.04 (0.789)	0.1 (0.469)	-0.03 (0.845)	-0.04 (0.751)	-0.04 (0.785)
	suicide seeking	-0.17 (0.208)	0.16 (0.223)	-0.02 (0.889)	0.07 (0.619)	-0.2 (0.144)	0.35 (0.01)	-0.25 (0.065)	0 (0.984)	0.09 (0.507)	-0.07 (0.598)	0.09 (0.501)	-0.04 (0.75)	0.09 (0.498)	0.06 (0.666)	-0.15 (0.264)	0 (0.982)	-0.07 (0.61)	-0.03 (0.84)	-0.04 (0.776)	0.05 (0.735)	0.12 (0.382)	-0.05 (0.704)	0.06 (0.642)	-0.07 (0.6)	0.09 (0.523)	-0.03 (0.816)	-0.03 (0.804)	0.09 (0.526)	-0.11 (0.424)
	suicide prevention	-0.03 (0.817)	-0.03 (0.796)	0.22 (0.098)	-0.11 (0.435)	-0.21 (0.113)	0.32 (0.017)	-0.15 (0.267)	-0.05 (0.711)	0.11 (0.424)	-0.02 (0.881)	0.03 (0.835)	0.04 (0.784)	-0.02 (0.91)	0.12 (0.368)	-0.25 (0.069)	-0.04 (0.782)	-0.09 (0.497)	-0.04 (0.795)	-0.02 (0.869)	0.1 (0.437)	-0.08 (0.533)	0.15 (0.265)	-0.06 (0.63)	0 (0.992)	-0.01 (0.945)	0.02 (0.858)	-0.04 (0.742)	0.07 (0.624)	0.02 (0.886)
	psychosis	0.03 (0.849)	-0.1 (0.46)	0.28 (0.036)	-0.23 (0.086)	-0.04 (0.772)	0.19 (0.154)	-0.24 (0.07)	0.02 (0.905)	0.02 (0.862)	-0.05 (0.686)	-0.01 (0.958)	0.01 (0.931)	0.14 (0.283)	0.19 (0.17)	-0.24 (0.075)	-0.04 (0.785)	0.01 (0.916)	-0.21 (0.116)	0.06 (0.675)	0.1 (0.448)	-0.04 (0.782)	-0.07 (0.582)	0.19 (0.156)	-0.14 (0.301)	0 (0.975)	0.03 (0.811)	0.01 (0.932)	-0.01 (0.959)	0.03 (0.842)
	mood / anxiety	-0.01 (0.933)	-0.03 (0.807)	0.21 (0.121)	-0.11 (0.407)	-0.2 (0.145)	0.28 (0.041)	-0.19 (0.165)	-0.17 (0.198)	0.16 (0.226)	0.03 (0.842)	-0.11 (0.399)	-0.01 (0.919)	0.02 (0.907)	0.18 (0.19)	-0.07 (0.593)	-0.02 (0.902)	-0.04 (0.791)	0.02 (0.884)	-0.02 (0.91)	0.11 (0.397)	-0.12 (0.371)	0.09 (0.512)	0.07 (0.579)	-0.13 (0.353)	0.03 (0.835)	0.02 (0.86)	-0.01 (0.92)	0.04 (0.748)	0.03 (0.82)
	economic stressor	0.05 (0.691)	-0.06 (0.643)	0.09 (0.52)	-0.03 (0.836)	-0.01 (0.944)	-0.05 (0.684)	0.1 (0.436)	-0.18 (0.179)	0.05 (0.703)	-0.07 (0.58)	-0.01 (0.965)	-0.18 (0.175)	0.17 (0.205)	0.26 (0.05)	0.6 (<0.001)	0.11 (0.402)	0.2 (0.137)	-0.03 (0.816)	-0.11 (0.416)	-0.02 (0.86)	-0.12 (0.386)	-0.03 (0.834)	-0.16 (0.248)	-0.16 (0.248)	-0.02 (0.862)	0 (0.992)	0.01 (0.94)	-0.07 (0.619)	-0.01 (0.945)
	social stressor	0.02 (0.886)	-0.06 (0.679)	0.22 (0.11)	-0.08 (0.571)	-0.13 (0.354)	0.24 (0.076)	-0.08 (0.574)	-0.19 (0.165)	0.2 (0.143)	-0.02 (0.854)	-0.06 (0.646)	0.01 (0.929)	-0.04 (0.785)	0.23 (0.082)	-0.15 (0.265)	-0.1 (0.48)	0 (0.983)	-0.13 (0.351)	0.06 (0.662)	0.06 (0.66)	-0.04 (0.76)	0.05 (0.733)	0.04 (0.749)	-0.09 (0.499)	0.08 (0.541)	-0.02 (0.858)	-0.06 (0.654)	0.04 (0.746)	0.03 (0.805)
New York DMA	suicide neutral	-0.03 (0.798)	0.03 (0.851)	0.06 (0.676)	0.03 (0.832)	-0.17 (0.203)	0.35 (0.01)	-0.22 (0.095)	-0.21 (0.125)	0.25 (0.068)	-0.05 (0.695)	0.17 (0.214)	-0.18 (0.173)	0.1 (0.457)	0.1 (0.445)	-0.19 (0.157)	0.15 (0.254)	-0.17 (0.203)	0.09 (0.493)	-0.16 (0.243)	0.17 (0.214)	-0.16 (0.225)	0.08 (0.554)	0.13 (0.322)	-0.11 (0.422)	0.16 (0.226)	-0.1 (0.478)	0.04 (0.779)	-0.06 (0.631)	0.07 (0.617)
	suicide seeking	-0.13 (0.331)	-0.03 (0.827)	0.22 (0.11)	0.02 (0.911)	-0.09 (0.503)	0.1 (0.458)	0.09 (0.505)	-0.41 (0.002)	0.3 (0.024)	0 (0.981)	0.04 (0.74)	-0.03 (0.827)	0.21 (0.118)	0.06 (0.643)	-0.26 (0.055)	0.15 (0.277)	-0.08 (0.544)	0.04 (0.764)	-0.06 (0.678)	0.07 (0.625)	-0.23 (0.094)	0.1 (0.445)	0.14 (0.293)	-0.11 (0.4)	0.07 (0.581)	-0.06 (0.676)	-0.05 (0.725)	0.1 (0.478)	-0.01 (0.94)
	suicide prevention	-0.07 (0.593)	0.1 (0.448)	-0.07 (0.623)	0.13 (0.317)	-0.27 (0.046)	0.2 (0.136)	-0.16 (0.245)	-0.01 (0.967)	0.12 (0.389)	-0.06 (0.64)	0.12 (0.37)	-0.03 (0.817)	0 (0.989)	-0.01 (0.937)	0.03 (0.824)	-0.11 (0.414)	-0.13 (0.321)	0.05 (0.702)	-0.07 (0.581)	0.24 (0.071)	-0.29 (0.03)	0.1 (0.459)	0.14 (0.284)	-0.17 (0.212)	0.12 (0.359)	-0.02 (0.853)	-0.02 (0.864)	0.02 (0.907)	0.06 (0.65)
	psychosis	-0.13 (0.35)	-0.09 (0.5)	0.14 (0.287)	-0.04 (0.773)	-0.02 (0.898)	0.03 (0.848)	-0.2 (0.147)	0.01 (0.917)	0.14 (0.295)	0.02 (0.903)	-0.02 (0.91)	0.11 (0.423)	-0.1 (0.474)	0.12 (0.371)	0.1 (0.471)	-0.08 (0.54)	-0.04 (0.777)	-0.11 (0.402)	0.01 (0.917)	0.26 (0.051)	-0.32 (0.018)	0.21 (0.116)	-0.1 (0.459)	-0.1 (0.436)	0.06 (0.668)	0 (0.977)	-0.06 (0.679)	0.02 (0.875)	0.07 (0.596)
	mood / anxiety	-0.05 (0.702)	-0.11 (0.396)	0.17 (0.215)	0.01 (0.965)	-0.22 (0.102)	0.14 (0.304)	-0.05 (0.727)	-0.2 (0.141)	0.12 (0.358)	0.03 (0.817)	0 (0.995)	-0.12 (0.372)	-0.02 (0.857)	0.14 (0.307)	0.22 (0.11)	-0.13 (0.329)	0.12 (0.355)	-0.07 (0.622)	-0.02 (0.901)	0.16 (0.227)	-0.13 (0.339)	0.07 (0.607)	0.16 (0.237)	-0.28 (0.041)	0.11 (0.397)	0.09 (0.498)	-0.13 (0.337)	0.04 (0.781)	0.06 (0.666)
	economic stressor	0.05 (0.708)	-0.04 (0.772)	0.1 (0.473)	-0.05 (0.738)	-0.08 (0.539)	-0.01 (0.965)	0.07 (0.583)	-0.23 (0.093)	0.13 (0.345)	-0.01 (0.964)	-0.04 (0.783)	-0.21 (0.117)	0.27 (0.049)	0.15 (0.257)	0.49 (<0.001)	0.04 (0.791)	0.14 (0.313)	-0.06 (0.669)	-0.1 (0.457)	0.07 (0.624)	-0.17 (0.202)	0.01 (0.954)	-0.11 (0.408)	-0.09 (0.504)	-0.04 (0.739)	0.04 (0.785)	-0.05 (0.695)	-0.07 (0.593)	0.07 (0.586)
	social stressor	0.06 (0.642)	-0.1 (0.449)	0.11 (0.41)	-0.02 (0.87)	-0.05 (0.722)	0 (0.998)	-0.01 (0.916)	-0.06 (0.645)	0.04 (0.79)	0.13 (0.323)	-0.09 (0.486)	-0.02 (0.888)	0 (0.985)	0.05 (0.704)	0.07 (0.603)	-0.13 (0.318)	0.09 (0.481)	-0.13 (0.344)	0.03 (0.815)	0.06 (0.679)	-0.02 (0.889)	0.05 (0.724)	0.09 (0.51)	-0.17 (0.22)	0.02 (0.858)	0.1 (0.478)	-0.14 (0.297)	0.09 (0.503)	0.03 (0.829)

**Time at home**		**Weekly lag**																												
**Region**	**Term Category**	-14	-13	-12	-11	-10	-9	-8	-7	-6	-5	-4	-3	-2	-1	0	1	2	3	4	5	6	7	8	9	10	11	12	13	14
National	suicide neutral	-0.13 (0.344)	-0.1 (0.461)	0.12 (0.36)	0.06 (0.669)	-0.02 (0.854)	-0.14 (0.299)	0 (0.986)	0.09 (0.488)	-0.01 (0.961)	0.08 (0.532)	-0.17 (0.202)	-0.06 (0.633)	0.04 (0.793)	0.05 (0.731)	0.18 (0.173)	0.06 (0.68)	-0.03 (0.824)	0.01 (0.944)	-0.02 (0.864)	0.09 (0.482)	-0.01 (0.943)	-0.07 (0.63)	0.02 (0.875)	-0.05 (0.706)	0.02 (0.909)	0.01 (0.958)	-0.02 (0.864)	0.03 (0.825)	-0.06 (0.635)
	suicide seeking	-0.15 (0.26)	-0.04 (0.745)	0.11 (0.405)	0.04 (0.753)	-0.04 (0.745)	-0.19 (0.17)	0 (0.986)	0.11 (0.408)	0.06 (0.665)	0.06 (0.648)	-0.29 (0.03)	-0.02 (0.863)	0 (0.991)	0.11 (0.434)	0.37 (0.006)	0.13 (0.343)	-0.01 (0.93)	-0.04 (0.74)	-0.02 (0.873)	0.14 (0.294)	0.04 (0.789)	-0.05 (0.715)	-0.05 (0.685)	-0.13 (0.345)	0.07 (0.624)	-0.02 (0.908)	-0.01 (0.952)	0.05 (0.722)	-0.08 (0.54)
	suicide prevention	-0.01 (0.926)	-0.01 (0.914)	0.07 (0.628)	-0.01 (0.929)	-0.03 (0.802)	-0.05 (0.729)	0.04 (0.78)	0.05 (0.692)	0.03 (0.817)	0 (0.996)	-0.08 (0.535)	-0.12 (0.366)	-0.08 (0.536)	0.04 (0.783)	0.42 (0.002)	0.21 (0.117)	-0.05 (0.705)	-0.04 (0.765)	-0.07 (0.591)	0.16 (0.223)	0.03 (0.811)	-0.01 (0.968)	-0.1 (0.459)	-0.1 (0.458)	0.01 (0.96)	0.02 (0.862)	-0.04 (0.746)	0.08 (0.545)	-0.04 (0.764)
	psychosis	0.02 (0.9)	0.04 (0.781)	-0.02 (0.873)	-0.03 (0.851)	-0.08 (0.549)	-0.11 (0.394)	0.06 (0.671)	0.01 (0.938)	0.1 (0.453)	0.13 (0.338)	-0.15 (0.252)	-0.18 (0.185)	-0.06 (0.66)	0.28 (0.036)	0.52 (<0.001)	0.18 (0.193)	-0.08 (0.544)	-0.03 (0.796)	-0.03 (0.82)	0.14 (0.296)	0.07 (0.589)	-0.05 (0.684)	-0.06 (0.65)	-0.15 (0.272)	-0.03 (0.802)	0 (0.994)	0.06 (0.637)	0.04 (0.769)	-0.09 (0.523)
	mood / anxiety	0 (0.991)	-0.1 (0.446)	0.02 (0.895)	0.07 (0.58)	-0.03 (0.803)	-0.15 (0.282)	-0.11 (0.418)	-0.03 (0.832)	0.12 (0.384)	0.04 (0.756)	-0.08 (0.54)	-0.2 (0.139)	-0.05 (0.734)	0.18 (0.177)	0.53 (<0.001)	0.25 (0.069)	-0.02 (0.857)	-0.03 (0.799)	-0.04 (0.774)	0.14 (0.307)	0.05 (0.708)	0.01 (0.916)	-0.08 (0.53)	-0.16 (0.232)	-0.01 (0.932)	0.01 (0.939)	0 (0.999)	0.09 (0.515)	-0.08 (0.568)
	economic stressor	-0.12 (0.369)	-0.15 (0.261)	-0.01 (0.919)	0.34 (0.011)	-0.02 (0.891)	-0.35 (0.01)	-0.51 (<0.001)	0.01 (0.948)	0.32 (0.018)	0.24 (0.071)	0.13 (0.325)	0.08 (0.575)	0.22 (0.097)	0.3 (0.025)	0.3 (0.027)	0.13 (0.32)	0.11 (0.433)	-0.01 (0.949)	-0.08 (0.539)	-0.1 (0.461)	0 (0.978)	0.07 (0.599)	-0.07 (0.63)	-0.14 (0.291)	-0.07 (0.601)	-0.01 (0.963)	0.03 (0.822)	0.01 (0.944)	-0.09 (0.502)
	social stressor	-0.01 (0.933)	0 (0.976)	0.09 (0.52)	0.03 (0.827)	-0.09 (0.527)	-0.12 (0.367)	0 (0.977)	0.1 (0.459)	0.08 (0.552)	0 (0.98)	-0.14 (0.297)	-0.12 (0.38)	-0.02 (0.91)	0.13 (0.337)	0.51 (<0.001)	0.22 (0.107)	-0.02 (0.89)	-0.04 (0.755)	-0.05 (0.713)	0.13 (0.317)	0.07 (0.606)	-0.04 (0.791)	-0.08 (0.569)	-0.16 (0.227)	0.01 (0.938)	0 (0.983)	-0.01 (0.963)	0.07 (0.624)	-0.04 (0.762)
New York DMA	suicide neutral	0.07 (0.629)	-0.05 (0.689)	0.07 (0.624)	-0.03 (0.831)	0.06 (0.634)	0.01 (0.968)	-0.04 (0.753)	0.07 (0.585)	-0.26 (0.055)	0.25 (0.059)	0.04 (0.787)	-0.2 (0.145)	0.24 (0.078)	-0.09 (0.502)	-0.08 (0.536)	0.17 (0.2)	-0.15 (0.254)	0.09 (0.498)	-0.2 (0.139)	0.21 (0.12)	-0.15 (0.254)	0.03 (0.809)	0.13 (0.332)	-0.1 (0.481)	0.12 (0.37)	-0.01 (0.965)	-0.02 (0.904)	-0.03 (0.802)	0 (0.989)
	suicide seeking	0.04 (0.784)	-0.01 (0.961)	0 (0.984)	0.12 (0.394)	0.07 (0.624)	-0.1 (0.461)	0.02 (0.885)	-0.04 (0.758)	-0.15 (0.28)	0.21 (0.119)	0.15 (0.266)	-0.35 (0.009)	0.42 (0.002)	0.04 (0.752)	-0.29 (0.033)	0.25 (0.061)	-0.13 (0.337)	0 (0.997)	-0.08 (0.56)	0.23 (0.09)	-0.26 (0.058)	-0.02 (0.901)	0.16 (0.239)	-0.12 (0.391)	0.1 (0.476)	-0.02 (0.907)	-0.02 (0.866)	0 (0.983)	-0.03 (0.803)
	suicide prevention	-0.07 (0.584)	0.15 (0.255)	-0.06 (0.642)	-0.02 (0.862)	0 (0.978)	0.07 (0.623)	-0.11 (0.411)	0.01 (0.925)	-0.02 (0.858)	0 (0.973)	0.13 (0.34)	-0.04 (0.79)	-0.04 (0.745)	0.02 (0.865)	-0.07 (0.617)	0.13 (0.325)	-0.2 (0.147)	-0.08 (0.539)	0.06 (0.636)	0.11 (0.4)	-0.16 (0.223)	0.12 (0.356)	0 (0.976)	-0.05 (0.694)	0.07 (0.581)	0.04 (0.745)	-0.06 (0.631)	-0.03 (0.803)	0.09 (0.517)
	psychosis	0.05 (0.724)	-0.22 (0.105)	0.07 (0.601)	0.1 (0.471)	-0.03 (0.802)	-0.01 (0.928)	0 (0.986)	-0.2 (0.145)	0.1 (0.478)	0.09 (0.51)	0.02 (0.895)	0.05 (0.736)	0.01 (0.964)	-0.12 (0.383)	0.15 (0.282)	0.11 (0.425)	-0.14 (0.304)	-0.05 (0.721)	0.07 (0.615)	-0.12 (0.367)	0.2 (0.145)	0 (0.972)	-0.13 (0.343)	-0.05 (0.716)	-0.03 (0.808)	0.02 (0.874)	0.13 (0.343)	-0.16 (0.25)	0.08 (0.566)
	mood / anxiety	0.16 (0.242)	-0.14 (0.284)	-0.05 (0.737)	0.2 (0.148)	-0.17 (0.219)	0.04 (0.74)	-0.01 (0.927)	-0.11 (0.396)	-0.04 (0.74)	0.12 (0.379)	0.07 (0.593)	-0.15 (0.266)	0.06 (0.644)	0.03 (0.809)	0 (0.976)	0.19 (0.155)	-0.02 (0.878)	-0.1 (0.471)	0.04 (0.772)	-0.05 (0.713)	0.02 (0.886)	0.16 (0.23)	0.02 (0.907)	-0.14 (0.291)	0.08 (0.538)	0.01 (0.942)	0.04 (0.789)	-0.01 (0.954)	-0.03 (0.821)
	economic stressor	0 (0.995)	0.07 (0.618)	-0.01 (0.96)	0.04 (0.758)	0.04 (0.789)	-0.13 (0.333)	0.07 (0.605)	-0.02 (0.889)	-0.05 (0.707)	0.12 (0.365)	-0.06 (0.673)	-0.15 (0.272)	0.17 (0.207)	0.2 (0.135)	0.12 (0.366)	0.29 (0.029)	0.14 (0.287)	-0.21 (0.121)	-0.03 (0.843)	0.01 (0.921)	-0.26 (0.056)	0.23 (0.094)	-0.22 (0.103)	-0.08 (0.576)	-0.02 (0.856)	0.01 (0.924)	0.03 (0.85)	-0.04 (0.763)	-0.02 (0.88)
	social stressor	-0.02 (0.863)	0.02 (0.897)	0.11 (0.428)	-0.16 (0.229)	0.1 (0.457)	0 (0.971)	-0.09 (0.488)	0 (0.99)	0.03 (0.808)	-0.05 (0.724)	0.08 (0.564)	0.01 (0.94)	-0.02 (0.875)	-0.03 (0.827)	0.12 (0.359)	-0.12 (0.384)	0.13 (0.333)	-0.2 (0.137)	0.12 (0.382)	-0.04 (0.768)	0.01 (0.915)	0.01 (0.935)	0.17 (0.216)	-0.22 (0.105)	0.1 (0.46)	-0.04 (0.742)	0 (1)	-0.01 (0.943)	0.09 (0.501)
