# Supplementary material for: Associations between COVID-19 mobility restrictions and economic, mental health, and suicide-related concerns in the US using cellular phone GPS and Google search volume data
Source: PLoS One. 2021 Dec 22;16(12):e0260931. doi: 10.1371/journal.pone.0260931 (PMC8694413; doi:10.1371/journal.pone.0260931)
Supplement: S4 Table — P-value in parenthesis. (PDF) [file pone.0260931.s007.pdf]

Sensitivity Analyses: January 6, 2019 - January 23, 2021

Proportion of devices at home		Weekly lag																																		
**Region**	**Term Category**	-17	-16	-15	-14	-13	-12	-11	-10	-9	-8	-7	-6	-5	-4	-3	-2	-1	0	1	2	3	4	5	6	7	8	9	10	11	12	13	14	15	16	17
National	suicide neutral	0.06 (0.523)	-0.04 (0.654)	-0.02 (0.811)	-0.03 (0.749)	0.05 (0.638)	-0.03 (0.783)	-0.02 (0.814)	0.03 (0.759)	0.1 (0.304)	-0.1 (0.302)	-0.06 (0.552)	0.01 (0.943)	0.03 (0.748)	0.02 (0.806)	0.01 (0.929)	0 (0.995)	0 (0.988)	-0.16 (0.097)	0.19 (0.055)	-0.11 (0.257)	-0.04 (0.697)	-0.01 (0.908)	0.02 (0.852)	0.09 (0.367)	-0.06 (0.508)	-0.05 (0.581)	0.08 (0.427)	-0.06 (0.542)	0.1 (0.294)	-0.09 (0.362)	-0.02 (0.807)	-0.05 (0.599)	0.08 (0.394)	0.03 (0.794)	0.04 (0.684)
	suicide seeking	0 (0.976)	-0.01 (0.954)	-0.05 (0.634)	-0.05 (0.62)	0.07 (0.471)	-0.06 (0.511)	0 (0.972)	0.02 (0.862)	0.14 (0.148)	-0.16 (0.095)	-0.01 (0.914)	0.06 (0.519)	-0.12 (0.225)	0.06 (0.539)	0 (0.961)	-0.02 (0.817)	0.11 (0.249)	-0.15 (0.11)	0.04 (0.644)	-0.07 (0.462)	0.03 (0.774)	-0.14 (0.154)	0.02 (0.865)	0.21 (0.034)	-0.09 (0.335)	-0.07 (0.495)	0.15 (0.118)	-0.03 (0.778)	-0.04 (0.713)	-0.02 (0.802)	0 (0.983)	-0.08 (0.416)	0.14 (0.151)	0.03 (0.78)	-0.05 (0.58)
	suicide prevention	0.08 (0.437)	-0.01 (0.924)	-0.05 (0.63)	-0.04 (0.682)	0.03 (0.727)	0.02 (0.813)	-0.04 (0.653)	-0.1 (0.284)	0.22 (0.023)	-0.07 (0.475)	-0.02 (0.828)	-0.04 (0.643)	0.04 (0.643)	-0.03 (0.723)	0.05 (0.571)	-0.02 (0.804)	0.21 (0.032)	-0.33 (<0.001)	0 (0.97)	-0.03 (0.744)	0.04 (0.668)	-0.07 (0.494)	0.1 (0.313)	-0.13 (0.181)	0.06 (0.549)	-0.04 (0.695)	0.06 (0.536)	-0.05 (0.602)	0.22 (0.023)	-0.09 (0.351)	-0.06 (0.52)	-0.07 (0.459)	0.16 (0.103)	-0.2 (0.035)	0.09 (0.359)
	psychosis	0.05 (0.624)	-0.03 (0.791)	-0.01 (0.882)	0.01 (0.912)	-0.04 (0.689)	-0.01 (0.917)	-0.03 (0.766)	0.02 (0.831)	0.15 (0.114)	-0.12 (0.23)	-0.04 (0.708)	-0.08 (0.401)	-0.05 (0.605)	0.07 (0.44)	-0.09 (0.368)	0.12 (0.213)	0.19 (0.045)	-0.2 (0.043)	-0.06 (0.503)	0.05 (0.608)	0.03 (0.734)	-0.04 (0.668)	0.01 (0.949)	-0.07 (0.495)	0.06 (0.512)	0.04 (0.672)	-0.01 (0.932)	0 (0.978)	0.09 (0.346)	-0.09 (0.34)	-0.07 (0.447)	-0.04 (0.702)	0.2 (0.039)	-0.16 (0.098)	0.09 (0.342)
	mood / anxiety	0.02 (0.849)	0 (0.968)	-0.01 (0.89)	-0.03 (0.771)	0.07 (0.483)	-0.03 (0.775)	-0.02 (0.864)	-0.13 (0.185)	0.15 (0.122)	-0.1 (0.308)	-0.05 (0.57)	0.01 (0.949)	0.02 (0.813)	-0.05 (0.572)	-0.03 (0.74)	-0.03 (0.795)	0.28 (0.004)	-0.15 (0.128)	0.01 (0.893)	0 (0.982)	0.08 (0.42)	-0.08 (0.422)	0.12 (0.222)	-0.09 (0.337)	0.01 (0.954)	0.1 (0.291)	-0.03 (0.734)	-0.09 (0.359)	0.18 (0.058)	0 (0.969)	-0.08 (0.406)	-0.04 (0.701)	0.16 (0.088)	-0.2 (0.041)	0.05 (0.621)
	economic stressor	-0.06 (0.553)	0.09 (0.366)	-0.04 (0.696)	0.07 (0.463)	0.06 (0.567)	0.05 (0.586)	-0.14 (0.145)	-0.1 (0.291)	0.02 (0.806)	0.18 (0.057)	0.03 (0.788)	0.11 (0.254)	0.02 (0.8)	0 (0.982)	-0.09 (0.358)	0.15 (0.114)	0.33 (<0.001)	0.51 (<0.001)	0.21 (0.028)	0.17 (0.083)	0 (0.972)	-0.04 (0.661)	0.04 (0.683)	0.02 (0.811)	0.09 (0.331)	0.01 (0.91)	-0.04 (0.697)	0.04 (0.704)	0.07 (0.491)	0.05 (0.621)	-0.02 (0.817)	0 (0.981)	0.04 (0.712)	0.05 (0.59)	0.01 (0.943)
	social stressor	-0.01 (0.951)	0.05 (0.634)	-0.09 (0.34)	0.03 (0.74)	0.02 (0.798)	0 (0.97)	-0.03 (0.749)	-0.04 (0.708)	0.18 (0.061)	-0.06 (0.51)	-0.1 (0.295)	0.07 (0.477)	-0.01 (0.937)	-0.04 (0.692)	0.04 (0.711)	-0.05 (0.586)	0.24 (0.014)	-0.14 (0.14)	-0.02 (0.808)	-0.03 (0.78)	0.01 (0.917)	0 (0.994)	0.02 (0.832)	-0.02 (0.816)	0 (1)	0.03 (0.763)	0.05 (0.578)	0.02 (0.806)	0.08 (0.396)	-0.06 (0.54)	-0.02 (0.818)	-0.04 (0.658)	0.08 (0.419)	-0.04 (0.685)	-0.03 (0.743)
New York DMA	suicide neutral	0.08 (0.403)	-0.07 (0.495)	-0.06 (0.518)	-0.08 (0.428)	0.08 (0.417)	-0.01 (0.956)	-0.01 (0.914)	-0.06 (0.565)	0.24 (0.014)	-0.15 (0.12)	-0.25 (0.009)	0.18 (0.066)	0.07 (0.479)	0.05 (0.612)	-0.12 (0.202)	-0.03 (0.72)	0.17 (0.077)	-0.17 (0.087)	0.13 (0.176)	-0.14 (0.139)	0.07 (0.447)	-0.1 (0.322)	0.04 (0.694)	-0.06 (0.514)	0.08 (0.397)	0.08 (0.434)	-0.03 (0.737)	0.03 (0.782)	-0.05 (0.606)	-0.04 (0.646)	0.01 (0.91)	0 (0.973)	0.08 (0.388)	-0.03 (0.767)	0.1 (0.317)
	suicide seeking	-0.04 (0.648)	0 (0.995)	-0.05 (0.587)	-0.06 (0.533)	-0.05 (0.613)	0.11 (0.272)	0.08 (0.415)	-0.08 (0.435)	0.12 (0.216)	-0.03 (0.724)	-0.22 (0.022)	0.11 (0.249)	0.14 (0.144)	-0.04 (0.643)	-0.07 (0.479)	0.17 (0.086)	-0.01 (0.928)	-0.1 (0.281)	0.08 (0.437)	-0.04 (0.679)	0.05 (0.597)	-0.1 (0.304)	0.07 (0.486)	-0.11 (0.264)	0.05 (0.578)	0.11 (0.261)	-0.03 (0.728)	0.08 (0.393)	-0.13 (0.165)	-0.04 (0.659)	0.14 (0.156)	0.03 (0.755)	-0.09 (0.366)	0.04 (0.653)	0.01 (0.952)
	suicide prevention	0.04 (0.702)	0.02 (0.866)	-0.02 (0.852)	-0.02 (0.808)	0 (0.961)	-0.03 (0.781)	0.05 (0.622)	-0.14 (0.155)	0.12 (0.219)	-0.05 (0.606)	-0.06 (0.543)	0 (0.978)	0.03 (0.731)	0.04 (0.696)	-0.02 (0.834)	0.02 (0.845)	0.05 (0.601)	-0.09 (0.348)	-0.03 (0.736)	0 (0.997)	-0.04 (0.664)	0.02 (0.825)	0.12 (0.212)	-0.17 (0.082)	0.03 (0.719)	0.07 (0.443)	-0.14 (0.146)	0.12 (0.227)	0.2 (0.04)	-0.12 (0.225)	-0.13 (0.185)	0.09 (0.336)	0.1 (0.321)	-0.11 (0.27)	-0.05 (0.629)
	psychosis	0.04 (0.698)	-0.05 (0.625)	0 (0.968)	-0.13 (0.167)	0.05 (0.641)	0.05 (0.589)	-0.03 (0.725)	0 (0.991)	-0.04 (0.717)	0.04 (0.68)	-0.08 (0.412)	0.03 (0.775)	-0.01 (0.952)	0.05 (0.602)	0 (0.999)	-0.03 (0.774)	0.08 (0.395)	0.14 (0.134)	-0.19 (0.056)	0 (0.98)	-0.03 (0.78)	-0.02 (0.799)	0.17 (0.087)	-0.15 (0.11)	0.1 (0.284)	-0.06 (0.537)	0.01 (0.933)	0.03 (0.76)	0 (0.982)	0.02 (0.876)	-0.08 (0.425)	0.08 (0.387)	-0.03 (0.779)	0.05 (0.588)	-0.1 (0.296)
	mood / anxiety	0.09 (0.359)	0.01 (0.888)	-0.03 (0.757)	-0.01 (0.906)	-0.06 (0.567)	0.1 (0.322)	0.02 (0.828)	-0.24 (0.015)	0.11 (0.269)	0 (0.975)	-0.1 (0.288)	-0.01 (0.953)	0.12 (0.21)	0.01 (0.952)	-0.19 (0.052)	0.05 (0.63)	0.17 (0.076)	0.14 (0.156)	-0.02 (0.846)	0.11 (0.257)	-0.06 (0.525)	-0.02 (0.806)	0.17 (0.075)	-0.1 (0.318)	0.09 (0.357)	0.1 (0.288)	-0.26 (0.008)	0.06 (0.56)	0.21 (0.033)	-0.09 (0.364)	-0.06 (0.557)	0.01 (0.902)	0.09 (0.353)	-0.11 (0.27)	0.06 (0.54)
	economic stressor	0.07 (0.456)	0.04 (0.703)	0.07 (0.439)	0.11 (0.246)	0.08 (0.428)	0.1 (0.291)	-0.08 (0.381)	-0.08 (0.382)	0.09 (0.369)	0.17 (0.08)	-0.01 (0.933)	0.18 (0.062)	0.1 (0.309)	-0.02 (0.857)	-0.12 (0.206)	0.26 (0.008)	0.33 (<0.001)	0.5 (<0.001)	0.18 (0.061)	0.14 (0.144)	-0.01 (0.901)	-0.03 (0.757)	0.09 (0.328)	-0.01 (0.881)	0.1 (0.299)	0.02 (0.807)	-0.01 (0.958)	0.03 (0.791)	0.09 (0.33)	0.02 (0.799)	-0.01 (0.903)	0.07 (0.467)	-0.04 (0.66)	0.13 (0.186)	-0.03 (0.746)
	social stressor	0.08 (0.437)	0.01 (0.901)	-0.08 (0.438)	0.01 (0.897)	0.05 (0.572)	-0.07 (0.469)	0.03 (0.747)	-0.02 (0.836)	-0.01 (0.934)	0 (0.97)	-0.07 (0.471)	0.08 (0.385)	0.1 (0.294)	-0.13 (0.177)	0.04 (0.715)	-0.02 (0.829)	0.09 (0.349)	0.05 (0.636)	-0.03 (0.757)	0.04 (0.694)	-0.03 (0.774)	-0.05 (0.588)	0.1 (0.323)	-0.02 (0.851)	0.03 (0.723)	0.07 (0.455)	-0.09 (0.364)	0.05 (0.618)	0.16 (0.099)	-0.15 (0.113)	0.07 (0.46)	-0.05 (0.588)	0.08 (0.412)	0.01 (0.881)	-0.12 (0.23)

Time at home		Weekly lag																																		
**Region**	**Term Category**	-17	-16	-15	-14	-13	-12	-11	-10	-9	-8	-7	-6	-5	-4	-3	-2	-1	0	1	2	3	4	5	6	7	8	9	10	11	12	13	14	15	16	17
National	suicide neutral	0.09 (0.336)	-0.12 (0.216)	-0.01 (0.922)	-0.04 (0.71)	0.03 (0.779)	0.01 (0.905)	0.01 (0.942)	0.03 (0.735)	0.04 (0.658)	-0.09 (0.348)	0.08 (0.423)	-0.13 (0.173)	0.16 (0.092)	-0.09 (0.352)	0.02 (0.869)	0.12 (0.205)	-0.09 (0.371)	0.03 (0.727)	-0.04 (0.676)	-0.07 (0.463)	0.09 (0.377)	-0.19 (0.047)	0.08 (0.412)	0 (0.992)	0.04 (0.704)	-0.01 (0.898)	-0.03 (0.719)	0.12 (0.208)	0.05 (0.599)	-0.09 (0.366)	0.09 (0.328)	-0.24 (0.014)	0.22 (0.025)	-0.12 (0.197)	0.09 (0.373)
	suicide seeking	0.1 (0.305)	-0.15 (0.123)	0 (0.975)	-0.01 (0.901)	0.07 (0.495)	0 (0.962)	0 (0.961)	0.07 (0.486)	0.04 (0.673)	-0.07 (0.467)	0.02 (0.835)	-0.14 (0.15)	0.21 (0.029)	-0.12 (0.221)	0.05 (0.598)	0.12 (0.233)	-0.07 (0.501)	0.02 (0.842)	-0.05 (0.618)	-0.11 (0.26)	0.07 (0.498)	-0.2 (0.044)	0.06 (0.539)	0.05 (0.592)	0.07 (0.45)	-0.06 (0.51)	-0.05 (0.609)	0.24 (0.012)	-0.02 (0.799)	-0.04 (0.644)	0.1 (0.319)	-0.27 (0.005)	0.13 (0.192)	-0.02 (0.837)	0.03 (0.722)
	suicide prevention	-0.01 (0.913)	-0.08 (0.398)	0.09 (0.327)	-0.05 (0.626)	-0.01 (0.957)	0.04 (0.704)	0.04 (0.683)	-0.01 (0.954)	0.05 (0.607)	-0.14 (0.152)	0.09 (0.347)	-0.07 (0.455)	0.12 (0.232)	-0.06 (0.552)	0.06 (0.526)	0.15 (0.109)	0.01 (0.894)	-0.25 (0.01)	0.05 (0.579)	-0.15 (0.125)	0.14 (0.158)	-0.3 (0.002)	0.04 (0.677)	0.03 (0.722)	0.11 (0.27)	-0.07 (0.476)	0.1 (0.307)	0.09 (0.361)	0.18 (0.067)	-0.11 (0.275)	0.04 (0.648)	-0.07 (0.447)	-0.04 (0.649)	-0.03 (0.738)	-0.05 (0.61)
	psychosis	-0.02 (0.852)	-0.05 (0.622)	0.04 (0.651)	0.02 (0.867)	-0.01 (0.928)	0.04 (0.644)	0.03 (0.786)	-0.01 (0.908)	-0.05 (0.579)	0.01 (0.913)	0.02 (0.822)	-0.18 (0.06)	0.08 (0.389)	-0.01 (0.956)	0.16 (0.09)	0.02 (0.844)	0.01 (0.877)	-0.12 (0.211)	0.11 (0.267)	-0.17 (0.074)	0.02 (0.867)	0 (0.968)	-0.12 (0.232)	0.1 (0.284)	0 (0.965)	-0.03 (0.757)	0.06 (0.543)	0.12 (0.205)	-0.05 (0.577)	-0.01 (0.935)	-0.06 (0.53)	-0.01 (0.881)	-0.08 (0.419)	0.02 (0.825)	0 (0.988)
	mood / anxiety	0.02 (0.808)	-0.08 (0.425)	0.05 (0.6)	0.15 (0.119)	-0.03 (0.757)	0.05 (0.589)	0.07 (0.468)	-0.04 (0.644)	0.02 (0.871)	-0.15 (0.123)	-0.01 (0.881)	-0.08 (0.405)	0.07 (0.465)	-0.07 (0.472)	0.03 (0.752)	0.13 (0.182)	0.06 (0.519)	-0.12 (0.204)	0.1 (0.307)	-0.09 (0.357)	0.11 (0.248)	-0.19 (0.044)	-0.03 (0.774)	0.09 (0.338)	0.06 (0.523)	-0.08 (0.434)	0.04 (0.682)	0.11 (0.235)	0.04 (0.701)	-0.06 (0.55)	0.04 (0.669)	-0.09 (0.339)	-0.02 (0.843)	-0.04 (0.701)	-0.08 (0.42)
	economic stressor	0.1 (0.319)	0.04 (0.69)	-0.14 (0.155)	0.13 (0.172)	0.11 (0.242)	0.12 (0.22)	-0.03 (0.796)	-0.1 (0.313)	-0.01 (0.928)	-0.07 (0.452)	-0.19 (0.047)	-0.15 (0.112)	-0.23 (0.018)	-0.14 (0.141)	-0.1 (0.316)	0.21 (0.031)	0.31 (0.001)	0.34 (<0.001)	0.29 (0.003)	0.28 (0.003)	0.07 (0.485)	-0.06 (0.546)	-0.2 (0.038)	-0.1 (0.314)	-0.04 (0.664)	-0.15 (0.129)	-0.15 (0.11)	-0.07 (0.493)	0.01 (0.927)	0.05 (0.584)	0.04 (0.642)	-0.02 (0.83)	-0.03 (0.793)	-0.02 (0.868)	0 (0.971)
	social stressor	0.04 (0.653)	-0.1 (0.286)	-0.03 (0.761)	0.09 (0.328)	-0.01 (0.914)	0.08 (0.437)	0.01 (0.948)	0.02 (0.873)	0.07 (0.488)	-0.1 (0.322)	0 (0.986)	-0.07 (0.498)	0.02 (0.81)	-0.04 (0.671)	0.08 (0.434)	0.14 (0.158)	0.02 (0.865)	-0.12 (0.203)	0.01 (0.905)	-0.09 (0.363)	0.08 (0.394)	-0.29 (0.002)	-0.01 (0.884)	0.18 (0.06)	-0.04 (0.67)	0.04 (0.704)	-0.02 (0.856)	0.2 (0.042)	0.05 (0.636)	-0.05 (0.607)	0 (0.97)	-0.02 (0.854)	-0.08 (0.414)	-0.02 (0.853)	-0.05 (0.64)
New York DMA	suicide neutral	0.12 (0.216)	-0.17 (0.075)	-0.01 (0.898)	0.03 (0.761)	0.02 (0.872)	0.04 (0.642)	-0.04 (0.663)	0.02 (0.874)	0.06 (0.516)	-0.04 (0.654)	0.03 (0.746)	-0.27 (0.006)	0.29 (0.003)	-0.02 (0.849)	-0.08 (0.394)	0.12 (0.225)	-0.08 (0.394)	0.03 (0.741)	0.05 (0.64)	-0.13 (0.188)	0.11 (0.259)	-0.2 (0.038)	0.13 (0.182)	-0.07 (0.447)	0.1 (0.318)	-0.06 (0.564)	0.02 (0.812)	0.03 (0.769)	0.04 (0.664)	-0.01 (0.948)	0 (0.98)	-0.09 (0.345)	0.08 (0.388)	0.03 (0.736)	-0.01 (0.912)
	suicide seeking	0.06 (0.559)	-0.08 (0.4)	0.04 (0.669)	-0.05 (0.611)	0.02 (0.86)	0.06 (0.55)	0.04 (0.669)	-0.03 (0.743)	0 (0.961)	0.11 (0.26)	-0.2 (0.035)	-0.04 (0.644)	0.14 (0.154)	0.04 (0.7)	-0.03 (0.72)	0.08 (0.423)	0.08 (0.425)	-0.13 (0.182)	0.12 (0.201)	-0.09 (0.35)	-0.03 (0.726)	-0.1 (0.306)	0.15 (0.128)	-0.12 (0.231)	0 (0.971)	0.03 (0.793)	-0.04 (0.663)	0.09 (0.35)	0 (0.988)	0.02 (0.845)	0.04 (0.686)	-0.16 (0.089)	0.15 (0.111)	-0.01 (0.882)	-0.04 (0.682)
	suicide prevention	-0.03 (0.752)	-0.01 (0.936)	0.05 (0.597)	-0.02 (0.818)	-0.02 (0.86)	0.04 (0.677)	-0.02 (0.852)	0 (0.97)	0 (0.972)	-0.03 (0.754)	-0.05 (0.618)	0.03 (0.73)	0.02 (0.863)	0.03 (0.744)	-0.01 (0.89)	0.1 (0.284)	-0.01 (0.906)	-0.14 (0.143)	0.06 (0.529)	-0.06 (0.537)	-0.03 (0.775)	-0.06 (0.517)	0 (0.998)	0.01 (0.887)	0.1 (0.324)	-0.04 (0.642)	-0.07 (0.494)	0.11 (0.246)	0.08 (0.38)	0.11 (0.24)	-0.2 (0.036)	0.07 (0.457)	-0.05 (0.595)	0.06 (0.541)	-0.13 (0.194)
	psychosis	0.05 (0.595)	-0.09 (0.365)	-0.01 (0.895)	-0.01 (0.909)	-0.07 (0.446)	0.05 (0.6)	0.08 (0.385)	-0.1 (0.295)	-0.01 (0.912)	0.05 (0.624)	-0.04 (0.665)	-0.05 (0.617)	0.04 (0.701)	0.11 (0.267)	-0.02 (0.814)	0.1 (0.283)	-0.16 (0.095)	0.18 (0.068)	-0.05 (0.582)	-0.03 (0.762)	-0.07 (0.443)	0.07 (0.452)	-0.17 (0.072)	0.23 (0.019)	-0.09 (0.372)	-0.09 (0.344)	0.03 (0.794)	0.1 (0.313)	-0.12 (0.225)	0.1 (0.324)	0.04 (0.662)	-0.15 (0.121)	0.03 (0.791)	0.11 (0.274)	-0.13 (0.172)
	mood / anxiety	0.15 (0.113)	-0.05 (0.592)	0.01 (0.933)	0.12 (0.204)	-0.17 (0.074)	0.07 (0.459)	0.15 (0.127)	-0.19 (0.055)	0.02 (0.836)	-0.02 (0.816)	-0.13 (0.174)	-0.01 (0.937)	0.13 (0.194)	-0.05 (0.579)	-0.08 (0.43)	0.06 (0.534)	0.09 (0.355)	-0.04 (0.647)	0.18 (0.061)	-0.02 (0.798)	-0.05 (0.612)	0.07 (0.45)	-0.15 (0.133)	0.12 (0.228)	0.05 (0.59)	-0.03 (0.792)	-0.07 (0.468)	-0.05 (0.592)	0.04 (0.708)	0.14 (0.144)	-0.14 (0.135)	0.03 (0.783)	-0.06 (0.507)	0 (0.988)	-0.03 (0.743)
	economic stressor	0.17 (0.083)	0.03 (0.731)	-0.08 (0.419)	0.01 (0.881)	0.13 (0.188)	0.02 (0.835)	0.01 (0.901)	-0.1 (0.313)	-0.11 (0.264)	0 (0.959)	-0.06 (0.513)	-0.09 (0.337)	0.04 (0.675)	-0.1 (0.316)	-0.13 (0.195)	0.17 (0.08)	0.25 (0.01)	0.22 (0.021)	0.33 (<0.001)	0.16 (0.099)	-0.12 (0.213)	-0.05 (0.576)	-0.04 (0.703)	-0.19 (0.054)	0.13 (0.171)	-0.16 (0.102)	-0.07 (0.473)	-0.05 (0.579)	0.01 (0.908)	0.01 (0.89)	-0.03 (0.746)	-0.02 (0.873)	-0.1 (0.296)	0.1 (0.29)	-0.1 (0.294)
	social stressor	0.07 (0.461)	-0.09 (0.336)	0.05 (0.609)	-0.02 (0.874)	-0.01 (0.922)	0.11 (0.264)	-0.09 (0.332)	0.04 (0.712)	0.03 (0.783)	-0.15 (0.109)	0.09 (0.366)	-0.03 (0.746)	0.03 (0.779)	-0.04 (0.711)	0.08 (0.431)	-0.02 (0.832)	0.07 (0.491)	0.01 (0.949)	-0.12 (0.209)	0.15 (0.128)	-0.13 (0.164)	0 (0.968)	-0.05 (0.576)	0.04 (0.671)	0.12 (0.226)	-0.08 (0.419)	-0.01 (0.882)	0.08 (0.401)	0 (0.985)	0.1 (0.317)	-0.13 (0.183)	0 (0.981)	0.05 (0.624)	-0.09 (0.33)	0.06 (0.533)
