# Supplementary material for: Associations between COVID-19 mobility restrictions and economic, mental health, and suicide-related concerns in the US using cellular phone GPS and Google search volume data
Source: PLoS One. 2021 Dec 22;16(12):e0260931. doi: 10.1371/journal.pone.0260931 (PMC8694413; doi:10.1371/journal.pone.0260931)
Supplement: S5 Table — ARIMA = autoregressive integrated moving average model. Non-seasonal ARIMA parameters = (p,d,q). p = autoregressive model order, d = degree of differencing, q = moving average model order. (PDF) [file pone.0260931.s008.pdf]

Time Period	Location	Mobility Indicator	Fitted Model
1/5/2020 - 1/23/2021	National	Time at home	ARIMA(2,0,2)
		Proportion of devices completely at home	ARIMA(2,0,0)
	NYC DMA	Time at home	ARIMA(2,0,0)
		Proportion of devices completely at home	ARIMA(2,0,0)
1/6/2019 - 1/23/2021	National	Time at home	ARIMA(1,1,0)
		Proportion of devices completely at home	ARIMA(2,1,0)
	NYC DMA	Time at home	ARIMA(1,1,1)
		Proportion of devices completely at home	ARIMA(2,0,1)
